# A Syx-RhoA-Dia1 signaling axis regulates cell cycle progression, DNA damage, and therapy resistance in glioblastoma

**DOI:** 10.1172/jci.insight.157491

**Published:** 2023-07-10

**Authors:** Wan-Hsin Lin, Ryan W. Feathers, Lisa M. Cooper, Laura J. Lewis-Tuffin, Jiaxiang Chen, Jann N. Sarkaria, Panos Z. Anastasiadis

**Affiliations:** 1 Department of Cancer Biology, Mayo Clinic, Jacksonville, Florida, USA.; 2 Department of Radiation Oncology, Mayo Clinic, Rochester, Minnesota, USA.

**Keywords:** Oncology, Brain cancer, Cell cycle, Drug therapy

## Abstract

Glioblastomas (GBM) are aggressive tumors that lack effective treatments. Here, we show that the Rho family guanine nucleotide exchange factor Syx promotes GBM cell growth both in vitro and in orthotopic xenografts derived from patients with GBM. Growth defects upon Syx depletion are attributed to prolonged mitosis, increased DNA damage, G2/M cell cycle arrest, and cell apoptosis, mediated by altered mRNA and protein expression of various cell cycle regulators. These effects are phenocopied by depletion of the Rho downstream effector Dia1 and are due, at least in part, to increased phosphorylation, cytoplasmic retention, and reduced activity of the YAP/TAZ transcriptional coactivators. Furthermore, targeting Syx signaling cooperates with radiation treatment and temozolomide (TMZ) to decrease viability in GBM cells, irrespective of their inherent response to TMZ. The data indicate that a Syx-RhoA-Dia1-YAP/TAZ signaling axis regulates cell cycle progression, DNA damage, and therapy resistance in GBM and argue for its targeting for cancer treatment.

## Graphical abstract

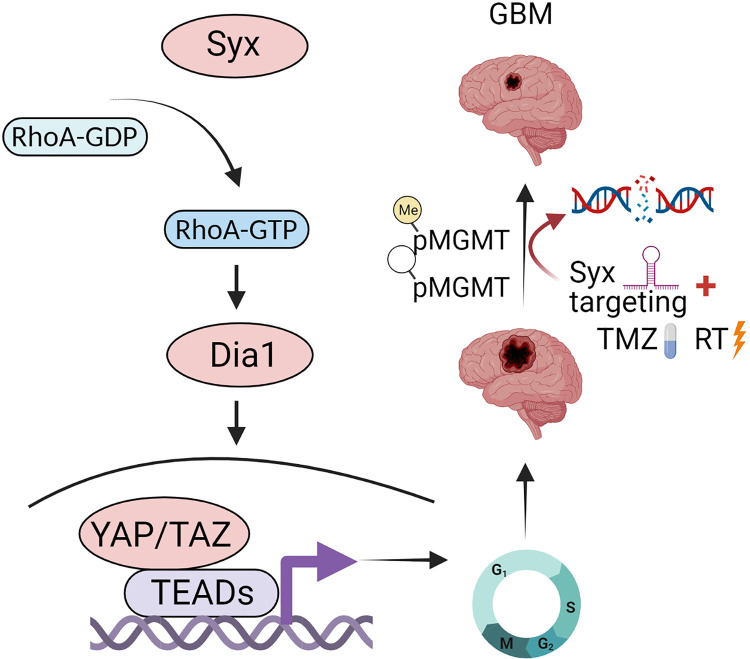

## Introduction

Glioblastomas (GBM) are malignant grade IV gliomas and are the most common primary brain tumors in adults. The hallmarks of GBM include aggressive growth and diffuse infiltration into adjacent brain parenchyma. Current standard of care for GBM involves maximal surgical resection, followed by concurrent chemoradiation therapy and adjuvant chemotherapy with temozolomide (TMZ) (1). Despite aggressive multimodal therapy, clinical outcomes for patients with GBM remain dismal. In the United States, the median survival for patients with GBM is about 15 months from initial diagnosis, and the 5-year survival rate is only about 5.5% (2). Major contributors to this poor outcome are the inevitable progression of the tumor, which is underlined by its infiltrative nature and the difficulty of complete surgical resection, tumor heterogeneity, and resistance to treatment. TMZ is a DNA alkylating agent that causes methylation of guanine and adenine nucleotides. The accumulation of unrepaired DNA lesions promotes G2/M cell cycle arrest and cell death, especially in tumors with low or no expression of O^6^-methylguanine-DNA-methytransferase (MGMT), a DNA repair protein (3). Tumors lacking MGMT promoter methylation and high MGMT expression account for more than 50% of GBM and show inferior responses to TMZ chemotherapy (4). Regardless of MGMT status, recurrence is universal in GBM, pointing to an urgent need for new therapeutic strategies.

The Rho family of small GTPases is composed of more than 20 members, including the most characterized RhoA, Rac1, and Cdc42. Rho GTPases regulate cytoskeletal remodeling, cell polarity, migration, gene expression, and cell cycle progression, coordinating diverse cellular functions. Rho guanine nucleotide exchange factors (GEFs) facilitate exchange of bound guanosine diphosphate (GDP) for guanosine triphosphate (GTP), promoting Rho GTPase activation (5). The active GTP-bound GTPases interact with downstream effectors to transduce signals to direct biological responses. Alterations of these GTPases and their regulators and effectors have been found in GBM and lead to aggressive disease (6).

Synectin-binding RhoA exchange factor (herein referred to as Syx, human gene symbol *PLEKHG5*) is a RhoA GEF that regulates junction integrity, cell migration, angiogenesis, and neural differentiation (7–11). Syx is expressed in endothelial cells, where its localization to the apical cell-to-cell junctions is regulated by the scaffold protein Mupp1 (and its paralogue Patj), VEGF signaling, and 14-3-3 protein binding (7, 12). Membrane-associated Angiomotin (Amot) forms a complex with Syx and Patj/Mupp1 to activate RhoA at the leading edge to direct endothelial cell migration (9, 11). Moreover, Syx is widely expressed in both conventional and patient-derived xenograft (PDX) GBM lines, promoting cell chemotaxis (8). The effects of Syx on junction integrity and cell migration are mediated by its ability to selectively induce the activation of mammalian Diaphanous formin (herein Dia1, human gene symbol *DIAPH1*) (7, 8).

RhoA and its effectors regulate gene expression and cell proliferation through multiple mechanisms, including the activation of the transcriptional cofactors Yes-associated protein 1 (YAP) and its paralog TAZ. Due to lack of a DNA-binding domain, YAP/TAZ associate with other transcription factors, such as TEA domain transcription factors (TEADs), to direct downstream responses to promote organ growth and cell transformation (13, 14). In GBM, YAP/TAZ expression and activity are thought to promote glioma aggressiveness (15, 16). Activation of Hippo kinases (MST1/2, LATS1/2) in response to extracellular cues, such as cell confluency and cell adhesion, results in phosphorylation, nuclear exclusion, and proteasomal degradation of YAP/TAZ (13, 14). Interestingly, expression of Amot and RhoA-mediated cytoskeleton remodeling can modulate YAP/TAZ activity via both Hippo kinase–dependent and –independent mechanisms (17, 18). Moreover, activation of Dia1 promotes the function of YAP and TAZ (19, 20).

Based on the roles of RhoA signaling in cell proliferation and transformation, we hypothesized that the previously established Syx-RhoA signaling pathway might regulate GBM cell growth in addition to promoting directed cell migration. Using conventional and xenograft GBM lines, we show here that downregulation of Syx-RhoA-Dia1 signaling in GBM cells results in cell cycle arrest, mitotic failure, DNA damage, and increased apoptosis in vitro as well as increased overall survival in orthotopic GBM xenograft–bearing mice. These effects are mediated, at least in part, by impaired activity of YAP/TAZ, leading to deregulated expression of key cell cycle regulators. Importantly, targeting this pathway augments cell responses to TMZ and radiation, pointing to its potential utility in GBM therapy.

## Results

### Depletion of Syx decreases GBM cell growth.

**Figure 1:**
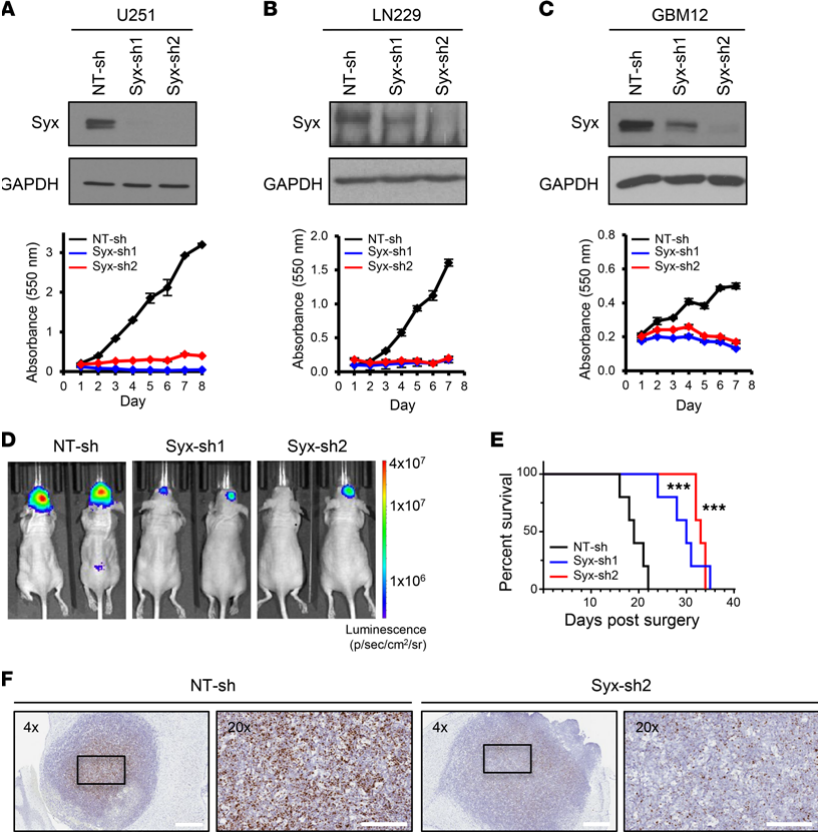
Depletion of Syx decreases GBM cell growth. (**A**–**C**) Immunoblot analysis of Syx and GAPDH in lysates from GBM conventional — U251 (**A**), LN229 (**B**) — and PDX —GBM12 (**C**) — cell lines transduced with indicated shRNAs (top). The same samples are shown with equal loading amounts run at different times (**A**) or in parallel (**B**). Cell viability over indicated time for each cell population was measured by the MTT assay (bottom). Shown are representative graphs with 3 technical replicates of 3–5 biological repeats. Graphs represent the mean ± SD. (**D**) Representative images of brain bioluminescence on day 19 after transplantation from intracranial xenografts derived from GBM12 cells expressing indicated shRNAs in immunocompromised mice. Luminescence in photons/sec/cm^2^/steradian units. (**E**) Kaplan-Meier survival curves of mice orthotopically transplanted with GBM12 cells transduced with indicated shRNAs. *n* = 5 mice per group. Log-rank test (***P < 0.001 for either Syx-sh1 or Syx-sh2 compared with NT-sh). (**F**) Expression of human Syx transcripts detected by RNAscope in situ hybridization in GBM12-derived xenografts expressing indicated shRNAs. Scale bar: 600 μm (4×), 200 μm (20×).

Increased proliferation and tumor cell dissemination are major factors in the poor prognosis of patients with GBM. We reported previously that Syx is expressed in GBM cells and is required for directed cell migration (8). To examine whether Syx also affects GBM cell growth, we utilized several conventional (U251, LN229, T98G) and PDX lines (GBM10, GBM12, GBM14). GBM10, GBM12, and GBM14 lines represent distinct transcriptional subtypes of GBM (GBM10 and GBM12 are mesenchymal, and GBM14 is classical subtype) and respond differently to TMZ due to their methylation status of the MGMT promoter (GBM12 cells are MGMT methylated and sensitive to TMZ, whereas GBM10 and GBM14 cells are MGMT unmethylated and TMZ resistant; ref. 21). Knockdown of *Syx* (*PLEKHG5*) by each of 2 nonoverlapping shRNAs resulted in a significant decrease in cell growth in tested GBM lines (Figure 1, A–C, and Supplemental Figure 1; supplemental material available online with this article; https://doi.org/10.1172/jci.insight.157491DS1). Next, we assessed the effect of *Syx* knockdown on GBM growth and overall survival in vivo using the orthotopic xenograft model. GBM12 cells were infected with lentiviruses expressing different shRNAs as well as with a luciferase-expressing virus. Infected cells were then implanted intracranially. Tumor growth was monitored using bioluminescence imaging. *Syx* knockdown resulted in decreased tumor growth, along with increased overall survival compared with the NT-shRNA (NT-sh) control (Figure 1, D and E). Expression of *Syx* RNA transcripts was assessed using RNAscope analysis to confirm the knockdown in vivo (Figure 1F). Overall, the data argue that depletion of Syx inhibits GBM cell growth in vitro and in vivo.

### Syx is required for GBM cell cycle progression and mitosis.

**Figure 2:**
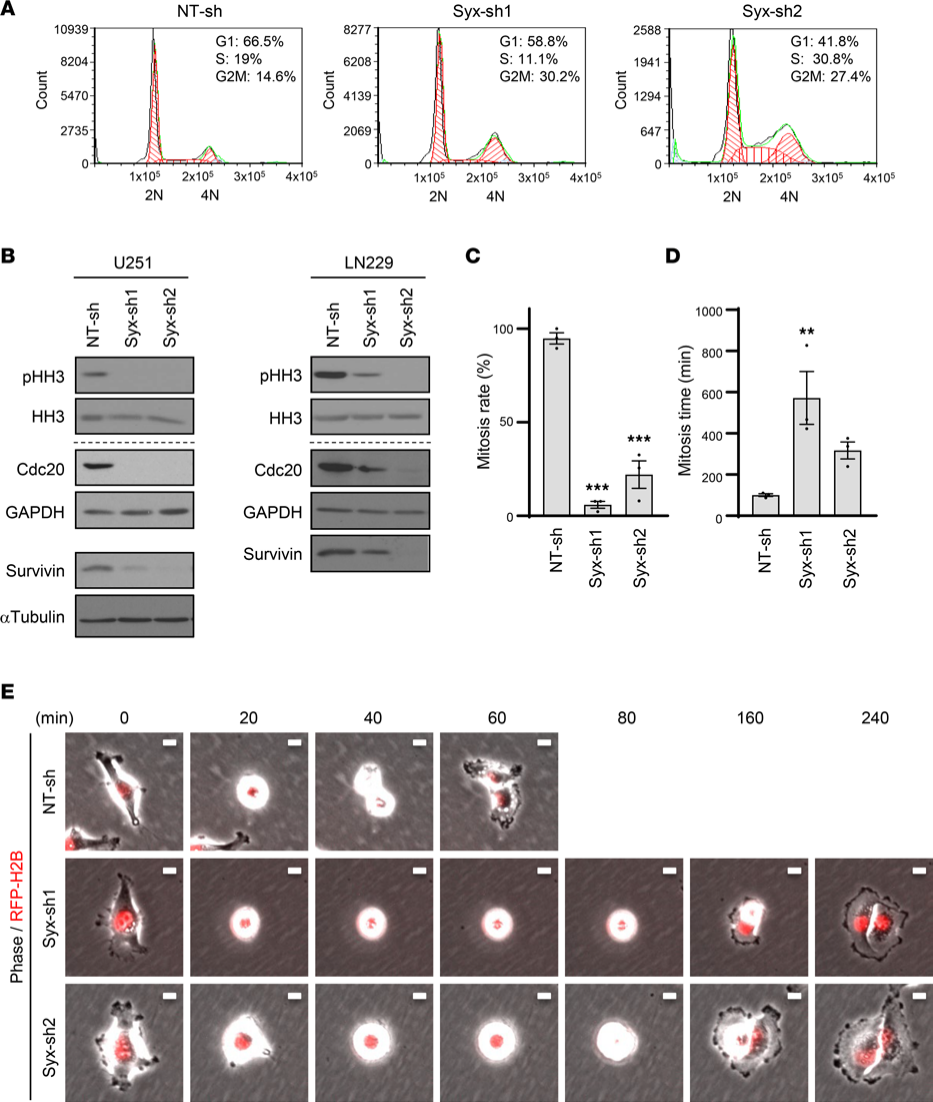
Syx is required for GBM cell cycle progression and mitosis. (**A**) Representative DNA fluorescence histograms and percentages of cells at different cell cycle phases (*n* = 3 biological repeats), as determined by propidium iodide-based DNA cell cycle analysis. DNA content (2n, 4n) is indicated. (**B**) Immunoblot analysis of mitotic markers (phosphorylated histone H3 at Ser10, pHH3), total histone 3 (HH3), and mitotic regulators (Cdc20, Survivin) in lysates of GBM cells (U251, LN229) expressing indicated shRNAs. Different biological samples are separated by dashed lines. The same biological samples run at different times are indicated by larger white space. pHH3 and HH3 blots were run in parallel. Survivin, Cdc20, and GAPDH were run in parallel (LN229). (**C**–**E**) Cell division was visualized and analyzed by 24-hour time-lapse imaging of U251 cells expressing indicated shRNAs and RFP-H2B. (**C**) Percentage of cells with successful cell division in each group (*n* > 100 cells per group). (**D**) The duration of mitosis (mitosis time) of cells that successfully underwent mitosis. Graphs (**C** and **D**) represent the mean ± SEM of 3 biological repeats. One-way ANOVA with Dunnett’s multiple-comparison test. **P < 0.01, ***P < 0.001. (**E**) Representative images (*n* = 3 experiments) of cells undergoing mitosis, acquired by time-lapse microscopy (time in minutes indicated above). Scale bar: 10 μm.

To investigate how Syx depletion affects cell growth, we examined its effects on cell cycle progression and cell apoptosis. Cell cycle analysis using flow cytometry and propidium iodide was performed in U251 cells expressing *Syx*-shRNAs (*Syx*-sh) or NT-sh control. Depletion of Syx resulted in cell cycle arrest at the G2/M phase compared with control (Figure 2A). To test whether this reflected a blockage of G2-to-M transition leading to reduced mitotic entry, we examined the level of phosphorylated histone H3 at Ser10 (pHH3), a well-accepted mitotic marker (22). Depletion of Syx resulted in reduced pHH3 levels in U251 and LN229 GBM cells, suggesting a defect in mitotic entry (Figure 2B). Moreover, the expression of 2 mitotic regulators, Cdc20 and Survivin, was also downregulated upon *Syx* knockdown (Figure 2B and Supplemental Figure 2). Cdc20 is a key cofactor of the anaphase-promoting complex (APC/C) ubiquitin ligase, which controls separation of sister chromatids in the metaphase-to-anaphase transition, as well as mitotic exit (23). During mitosis, Survivin, a member of the chromosome passenger complex (CPC), governs proper chromosome positioning and segregation (24). To directly examine cell division, we performed time-lapse imaging of cells expressing fluorescently labeled histone H2B to better visualize chromosomes (Figure 2, C–E, and Supplemental Videos 1–3). During the 24-hour imaging period, approximately 95% of NT-sh cells were able to enter and complete cell division (Figure 2C). Consistent with the reduced pHH3 levels (Figure 2B), only 6% of *Syx*-sh1 and 22% of *Syx*-sh2 cells were able to undergo successful cell mitosis (Figure 2C). Furthermore, NT-sh cells completed mitosis in approximately 1.7 hours, whereas the *Syx*-knockdown cells that were able to undergo mitosis required at least 5 hours (Figure 2D). Combined, the data suggest that Syx depletion results in 2 phenotypes, a predominant defect in mitotic entry, as well as prolonged mitosis.

### Syx depletion increases DNA damage.

**Figure 3:**
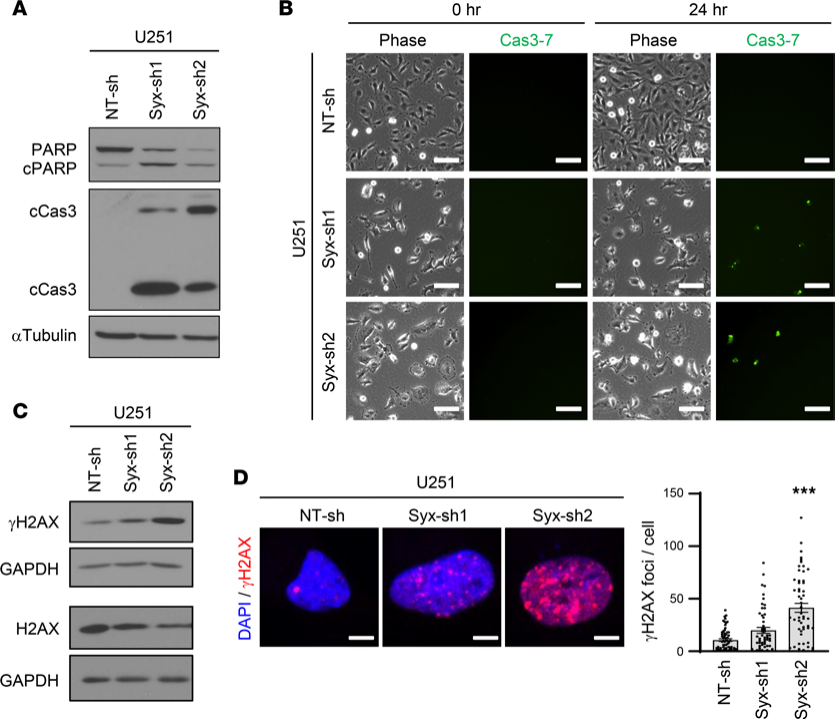
Syx depletion increases DNA damage. (**A**) Immunoblot analysis of apoptotic markers — cleaved caspase 3 (cCas3) and cleaved PARP (cPARP) — and α-tubulin in lysates of U251 cells transduced with Syx shRNAs. The same samples with equal loading were run in parallel. (**B**) Phase contrast (Phase) and corresponding fluorescence images of activated effector caspases (caspases-3/7) using a Cas3-7 probe (2 biological repeats). Shown are images acquired at time 0 and 24 hours after addition of the Cas-3/7 probe. Scale bar: 100 μm. (**C**) Immunoblot analysis of phosphorylated H2AX at Ser-139 (γH2AX), total H2AX, and GAPDH in lysates of U251 cells expressing indicated shRNAs. The same samples for H2AX and γH2AX blots with corresponding GAPDH loading controls were run at different times (indicated by larger white space). γH2AX and GAPDH blots were run in parallel. (**D**) Immunofluorescence staining of nucleus (DAPI, blue) and γH2AX foci (red) in U251 cells expressing indicated shRNAs. Representative images are shown (left). Scale bar: 5 μm. Bar graph (right) depicts the average ± SEM number of γH2AX foci per cell in U251 cells transduced with indicated shRNAs (*n* > 50 cells per group). One-way ANOVA with Dunnett’s multiple-comparison test. ***P < 0.001.

Prolongation of mitosis has been linked to double-stranded DNA damage and activation of the DNA damage response (DDR) (25, 26), in part through the activation of executioner caspases (caspase 3/7). Consistent with this, knockdown of *Syx* resulted in upregulation of cleaved caspase 3 and poly(ADP-ribose) polymerase (PARP), accompanied by downregulation of uncleaved PARP (Figure 3A). Additionally, incubation of U251 cells with a Cas3-7 biosensor, which becomes fluorescent upon activation of executioner caspases, indicated that more *Syx*-knockdown cells exhibit emission of fluorescence signal compared with control NT-sh cells (Figure 3B).

To test whether DNA damage is induced upon Syx depletion, we assessed the level of Ser139-phosphorylated Histone H2AX (known as γH2AX), a sensitive marker of double-strand breaks (DSBs) (27). Depletion of Syx increased both the phosphorylation of H2AX and the overall number of γH2AX nuclear foci in U251 cells (Figure 3, C and D). Importantly, similar results were also obtained in GBM10 and GBM12 PDX lines (Supplemental Figure 3, A–D).

Previously, we and others reported a crucial role of Syx in the regulation of endothelial cell junction stability, vascular permeability, and endothelial cell migration (7, 9, 11). To account for non–tumor-specific effects of inhibiting Syx, we assessed whether Syx silencing also induces DNA damage in primary human brain microvascular endothelial cells (HBMECs). In contrast to GBM cells, Syx depletion did not increase the number of γH2AX foci in HBMECs (Supplemental Figure 3, E and F).

### Syx regulates the expression of cyclins and cyclin-dependent kinase inhibitors.

**Figure 4:**
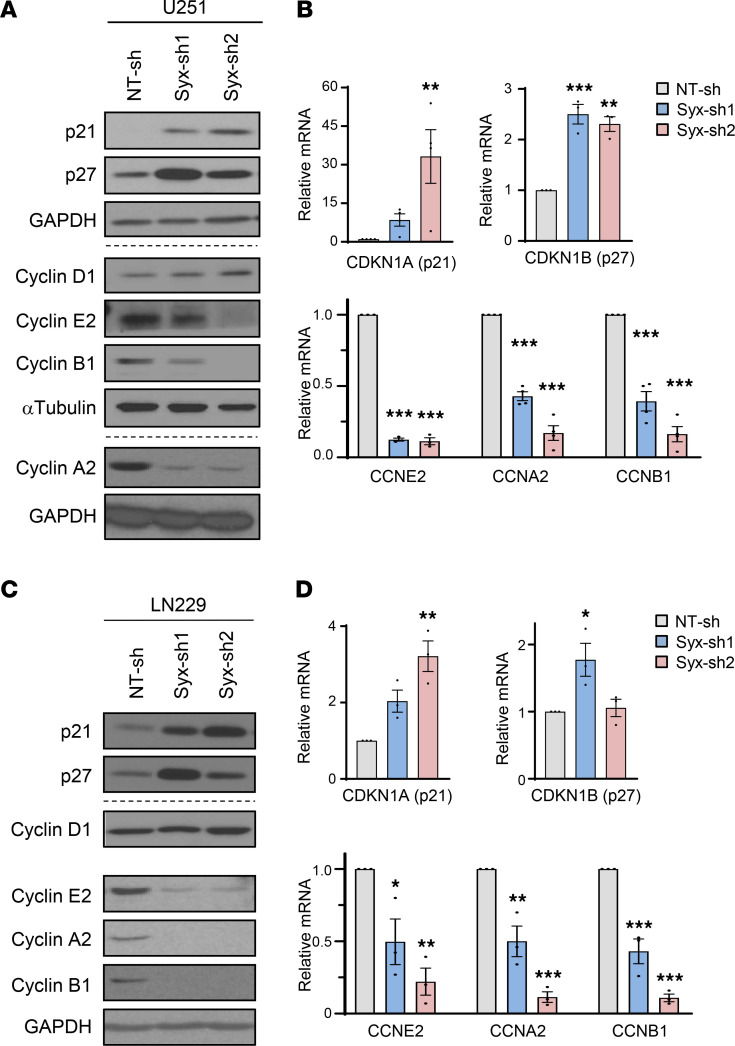
Syx regulates the expression of cyclins and cyclin-dependent kinase inhibitors. (**A**–**D**) Immunoblotting (**A** and **C**) and qPCR (**B** and **D**) analyses of the expression of p21 (CDKN1A), p27 (CDKN1B), Cyclin D1, Cyclin E2 (CCNE2), Cyclin A2 (CCNA2), and Cyclin B1 (CCNB1) in U251 (**A** and **B**) and LN229 (**C** and **D**) cells expressing indicated shRNAs, grown at subconfluency. For Western blots (**A** and **C**), different biological samples are separated by dashed lines. Samples from each blot set were run in parallel, except Cyclin A2 and the corresponding GAPDH from the same gel blot. The same samples for the Cyclin D1 blot with equal loading amounts as other Cyclins blots were run at different times (**C**, indicated by larger white space). See Figure 2B for the GAPDH loading control for p21 and p27 blots in **C**. Bar graphs (**B** and **D**) represent mean ± SEM of 3–4 biological replicates of relative mRNA expression of indicated genes normalized by GAPDH or β-actin. One-way ANOVA with Dunnett’s multiple-comparison test. *P < 0.05, **P < 0.01, ***P < 0.001.

The transition from one phase of the cell cycle to another is regulated by the coordinated action of cyclins, cyclin-dependent kinases (CDKs), and CDK inhibitors (CKIs) (28). Knockdown of *Syx* in either U251 or LN229 GBM cells resulted in upregulation of the CKIs p21^Cip1^ and p27^Kip1^, and downregulation of cyclin E2, cyclin A2, and cyclin B1 (Figure 4). Similar effects were also observed in all 3 GBM PDX lines used in this study (Supplemental Figure 4A). Using the CDK1 inhibitor RO-3306 to synchronize U251 cells in the G2 phase (28), the level of pHH3 in NT-sh cells increased as cells entered mitosis upon RO-3306 release, while the level of cyclin B1 progressively decreased as cells exited mitosis (Supplemental Figure 4B). Of note, despite being arrested in G2/M (Figure 2A), *Syx*-knockdown cells exhibited very low expression of cyclin B1 both before and after release from RO-3306 treatment (Supplemental Figure 4C). This result suggests that deregulation of cyclin B1 (and possibly other cell cycle regulators) upon Syx deficiency is not a consequence but likely a cause of cell cycle arrest.

To identify the underlying mechanism of cyclin and CKI deregulation in Syx-knockdown cells, we performed quantitative PCR (qPCR) to measure the RNA levels of these molecules. The data indicate that deregulation of cyclins and CKIs in U251 and LN229 cells occurs at the mRNA level (Figure 4, B and D). Interestingly, Cdc20 and Survivin transcripts were also downregulated upon *Syx* silencing (Supplemental Figure 2, A and B). The results support that Syx affects transcription to regulate cell cycle progression.

### Dia1 and YAP/TAZ signaling are downstream effectors of Syx.

**Figure 5:**
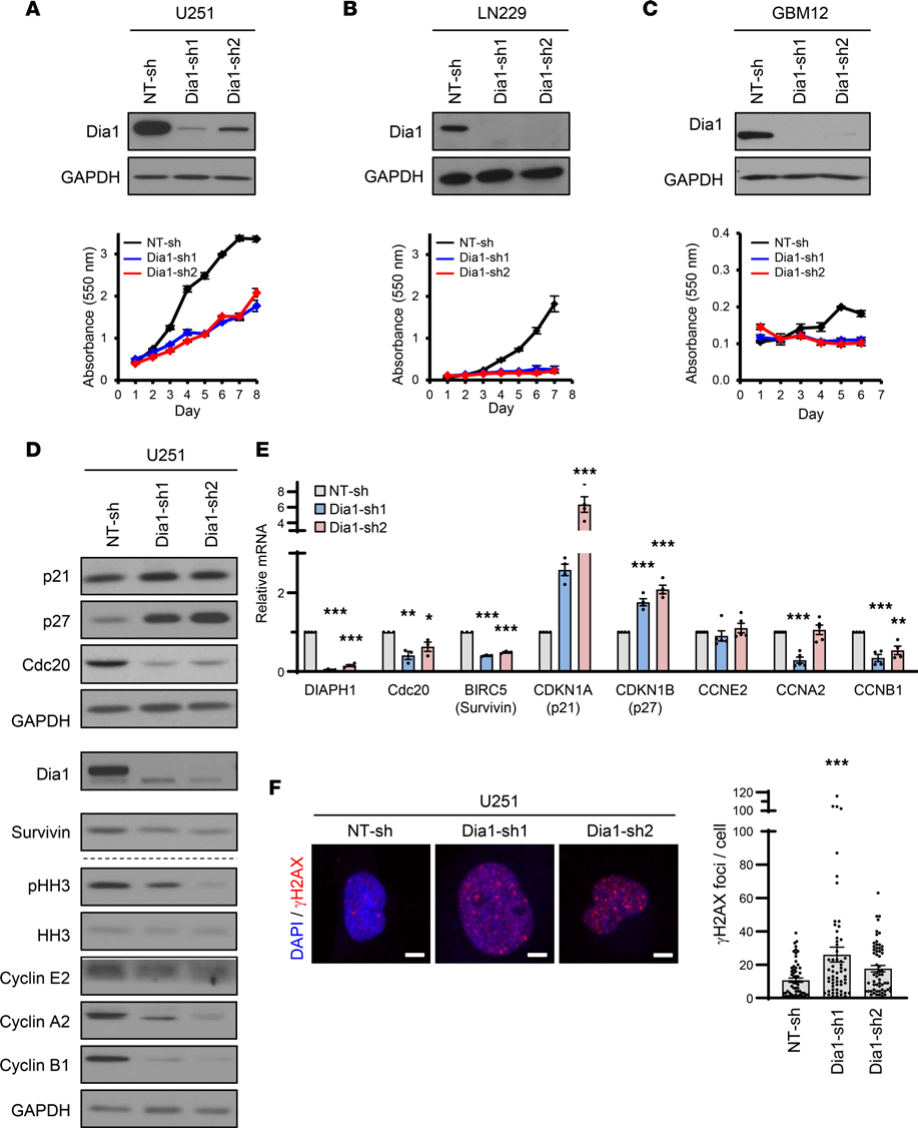
Dia1 is a downstream effector of Syx for the regulation of cell growth and gene expression. (**A**–**C**) Immunoblot analysis of Dia1 and GAPDH in lysates of U251 (**A**), LN229 (**B**), or GBM12 (**C**) cells transduced with *Dia1* shRNAs (top). Cell viability over the indicated time for each cell population was measured by the MTT assay (bottom). Shown are representative graphs (mean ± SD) of 3 biological repeats with 3 technical replicates each. (**D** and **E**) Immunoblot (**D**) and qPCR (**E**) analyses of phosphorylated histone 3 at Ser-10 (pHH3), total histone 3 (HH3), Cdc20, Survivin (BIRC5), p21 (CDKN1A), p27 (CDKN1B), Cyclin E2 (CCNE2), Cyclin A2 (CCNA2), and Cyclin B1 (CCNB1) in U251 cells expressing indicated shRNAs. Different biological samples are separated by dashed lines. Dia1 and Survivin blots were run at different times (indicated by larger white space). All other blots were run in parallel. Graph (**E**) represents the mean ± SEM of 3–5 biological replicates of relative mRNA expression of indicated genes normalized by GAPDH. (**F**) Representative images of immunofluorescence staining (left) show γH2AX (red) foci in the nucleus (DAPI, blue) of U251 cells expressing indicated shRNAs. Scale bar: 5 μm. Bar graph (right) depicts the average ± SEM number of γH2AX foci per cell in U251 cells transduced with indicated shRNAs (*n* > 60 cells per group). One-way ANOVA with Dunnett’s multiple-comparison test. *P < 0.05, **P < 0.01, ***P < 0.001.

To gain insight into the mechanism of Syx action, we investigated the involvement of potential downstream effectors of the Syx-RhoA signaling pathway. First, we focused on the role of the formin Dia1, a known downstream effector of Syx in the context of endothelial junction formation and GBM cell migration (7, 8). Similar to *Syx* knockdown, downregulation of *Dia1* (*Diaph1*) by each of 2 nonoverlapping shRNAs (7, 8) resulted in decreased cell growth in multiple GBM lines (U251, LN229, GBM12, GBM14) (Figure 5, A–C, and Supplemental Figure 5A). Moreover, expression of cyclins, CKIs, and mitosis regulators was deregulated at both mRNA and protein levels (Figure 5, D and E). Downregulation of pHH3 levels suggested a defect in mitotic entry (Figure 5D). Additionally, knockdown of *Dia1* increased DSBs as indicated by γH2AX immunofluorescence staining (Figure 5F). The ability of Dia1 to phenocopy Syx effects on the expression of cell cycle regulators, overall cell growth, and DNA damage argues that Dia1 is a key downstream effector of Syx growth-related signaling.

**Figure 6:**
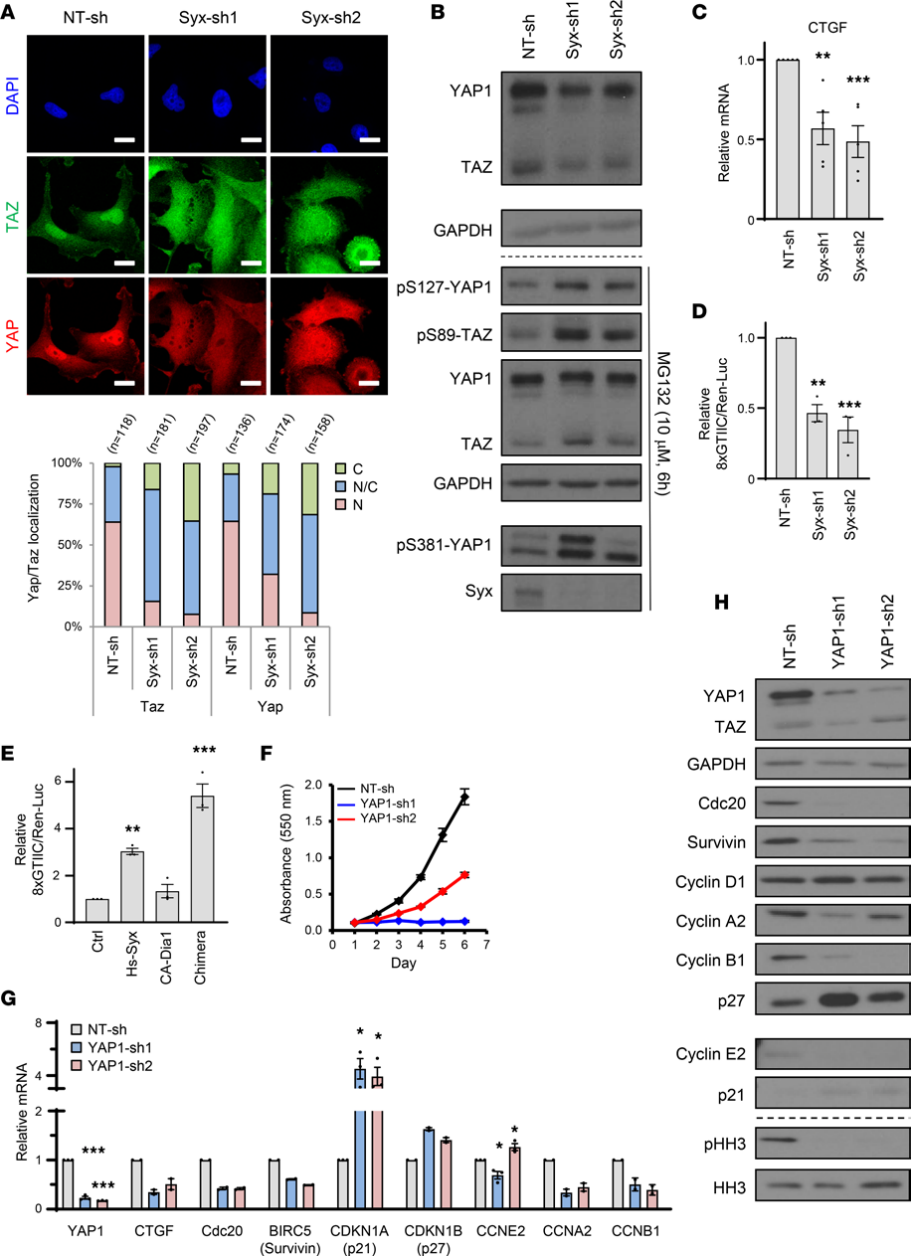
Dia1 and YAP/TAZ are downstream effectors of Syx. (**A**) Immunofluorescence images (top) of YAP (red) and TAZ (green) subcellular localization in U251 cells expressing *Syx* shRNAs. Scale bar: 15 μm. Staggered graphs (bottom) depict percentage of cells with YAP and TAZ in the cytosol (C), nucleus (N), or both (N/C). (**B**) Immunoblots showing total and phosphorylated YAP1/TAZ, Syx, and GAPDH in U251 cells treated with (bottom) or without (top) MG132. (**C**) qPCR analysis of relative mRNA levels of CTGF in U251 cells expressing indicated shRNAs. Graph represents the mean ± SEM of 5 biological replicates. (**D** and **E**) Graphs show relative luciferase activity of a YAP/TAZ responsive reporter (8xGTIIC) in U251 cells expressing indicated shRNAs (**D**) or expressing indicated constructs (**E**). Renilla luciferase activity (Ren-Luc) was used to normalize 8xGTIIC activity. Graphs represent mean ± SEM of 3 biological replicates. (**F**) Cell viability over indicated time for each cell population as measured by MTT assay (mean ± SD). Shown is representative of 3 biological replicates with 3 technical repeats each. (**G** and **H**) qPCR (mean ± SEM of 2–3 biological repeats) and immunoblot analyses of indicated mRNAs and corresponding proteins in U251 cells expressing indicated shRNAs. For Western blots (**B** and **H**), different biological samples are separated by dashed lines. The same biological samples run at different times are indicated by larger white space. All other blots were run in parallel. One-way ANOVA with Dunnett’s multiple-comparison test. *P < 0.05, ***P < 0.001.

Next, we focused on signaling pathways downstream of Syx-RhoA-Dia1 that could influence the expression of cell cycle regulators. One pathway by which RhoA drives transcription involves serum response factor (SRF) (29) and its coactivator myocardin-related transcription factor (MRTF), which couple changes in actin dynamics and cell shape to gene expression and cell growth (30). Another pathway by which both RhoA and Dia1 affect transcription is through the regulation of YAP/TAZ activity (19, 20). Interestingly, YAP/TAZ have been previously shown to regulate cell cycle progression (31, 32) and the expression of cyclins, CKIs, Survivin, and Cdc20 in other cell types, either directly or indirectly (32–34). Consistent with an involvement of YAP/TAZ in the Syx-RhoA-Dia1 signaling axis, silencing of either *Dia1* or *Syx* resulted in increased cytoplasmic localization of YAP/TAZ and concomitant decrease in nuclear localization relative to the NT-sh control (Figure 6A and Supplemental Figure 5, B–D). The cellular localization of YAP/TAZ is largely affected by its phosphorylation status, with phosphorylation at Ser-127 of YAP (Ser-89 in TAZ) being critical for its cytoplasmic sequestration via interaction with 14-3-3 proteins (35) and phosphorylation at Ser-381 of YAP (Ser-311 in TAZ) linked to its protein stability (36, 37). We thus examined how Syx affects YAP/TAZ stability and phosphorylation at these 2 residues. In Syx-knockdown cells, overall YAP/TAZ protein levels were downregulated (Figure 6B, top) and could be reversed when proteasome function was inhibited by MG132 (Figure 6B, bottom). Treatment of Syx-knockdown cells with MG132 also increased phosphorylation levels of YAP at S127 and S381, and TAZ at S89 (Figure 6B, bottom). Notably, γH2AX upregulation by Syx-knockdown could be suppressed by the expression of a nonphosphorylatable constitutively active (CA) mutant of YAP1 (YAP1-S127/381A; YAP1-AA) (Supplemental Figure 5E). The data suggest that Syx depletion promotes cytoplasmic retention and proteasomal degradation of YAP/TAZ by increasing their phosphorylation levels.

In agreement, expression of connective tissue growth factor (CTGF), a well-established direct target gene of YAP/TAZ(14), was downregulated by *Syx* silencing (Figure 6C). Moreover, knockdown of *Syx* resulted in downregulation of YAP/TAZ activity (Figure 6D and Supplemental Figure 5F), as measured by a YAP/TAZ-responsive luciferase reporter (GTIIC-Luc) (38). In contrast, ectopic expression of Syx resulted in increased transcriptional activity of YAP/TAZ (Figure 6E). Previously, we generated a chimera construct that consists of a CA Dia1 fragment (ΔN3; lacking the autoinhibitory Rho-binding domain) fused to a C-terminal Syx fragment containing the PDZ binding motif (Syx[C]) responsible for membrane recruitment of Syx (8). Expression of YFP-Dia1(ΔN3)-Syx(C), hereafter referred to as “chimera,” promotes Dia1-induced signaling events selectively at Syx-targeted membrane complexes and rescues polarity defects in Syx-depleted cells (8). Expression of this chimera in U251 cells increased YAP/TAZ activity by 5-fold, while expression of YFP-Dia1(ΔN3) failed to significantly increase YAP/TAZ activity (Figure 6E). These data suggest that localized activation of Syx-RhoA-Dia1 signaling promotes the nuclear localization and transcriptional activity of YAP/TAZ to affect gene expression. Confirming this hypothesis, *YAP1* silencing decreased cell growth in vitro and altered the expression of the same cell cycle regulators affected by knockdown of *Syx* and *Dia1* (Figure 6, F–H, and Supplemental Figure 5G).

While the above data indicate a key role for YAP/TAZ in the Syx-RhoA-Dia1 signaling axis, they do not exclude potential contributions from other RhoA-induced signaling pathways. Indeed, depletion of Syx in U251 cells reduced SRF/MRTF activity, both in control cells and under conditions of serum stimulation, as measured by an SRF/MRTF-response element luciferase reporter (SRF-RE) (Supplemental Figure 6A). The growth of GBM cells in vitro was also significantly suppressed by CCG-203971, a specific inhibitor of SRF/MRTF signaling (Supplemental Figure 6B).

### Targeting Syx-Dia1 signaling cooperates with TMZ and radiation treatment (RT).

**Figure 7:**
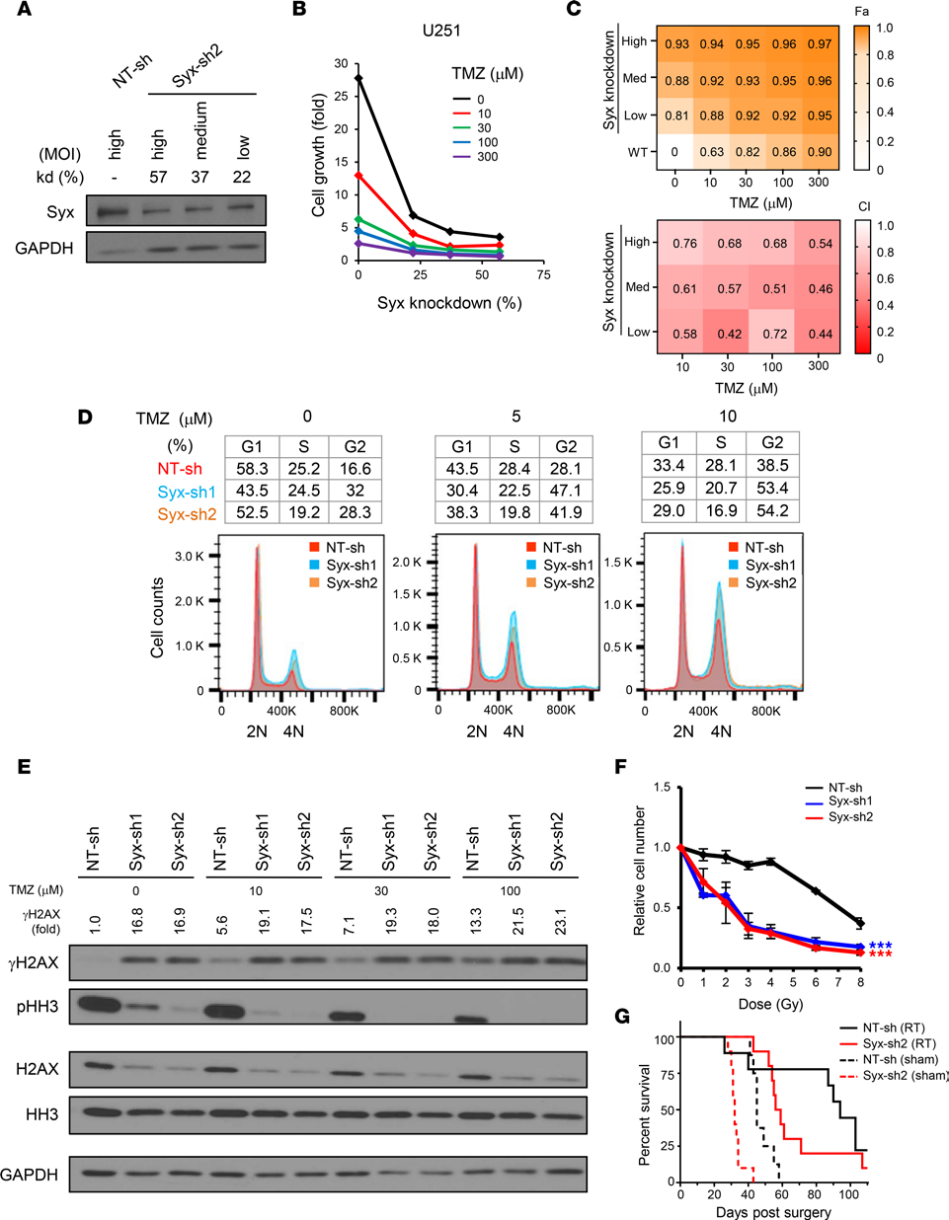
Syx-knockdown cooperates with temozolomide and radiation to suppress cell growth. (**A**) Immunoblot analysis of Syx in U251 cells transduced with Syx-sh2 expressing lentiviruses at different multiplicities of infection (MOI). Syx knockdown efficacy (kd %) relative to GAPDH expression is indicated. (**B**) Representative graph of 3 biological repeats depicts the growth of U251 cells exhibiting different degrees (%) of Syx knockdown (*x* axis) and treated with or without TMZ for 6 days. (**C**) Heatmaps depict average relative growth inhibition (top) and synergistic interaction (bottom) between Syx targeting and TMZ from 3 biological repeats. Fa, affected fraction; CI, combination index. Orange (top panel) indicates high Fa, which corresponds to high growth inhibition (top). Red (bottom panel) indicates low CI, corresponding to high synergy. (**D**) Representative DNA fluorescence histograms (top) and cell cycle distribution (bottom) of U251 cells expressing NT-sh (red), Syx-sh1 (cyan), or Syx-sh2 (orange) and treated with 0 μM, 5 μM, or 10μM TMZ (*n* = 3 experiments). (**E**) Immunoblot analysis of γH2AX, total H2AX, pHH3, total HH3, and GAPDH in lysates of U251 cells expressing indicated shRNAs and treated with TMZ for 4 days. γH2AX levels normalized to GAPDH and compared with the NT-sh nontreated control are indicated as γH2AX fold. The same biological samples run at different times are indicated by larger white space. All other blots were run in parallel. (**F**) Graph (mean ± SD, 2–3 biological repeats) shows the relative number of U251 cells expressing indicated shRNAs following treatment with different doses of radiation (Gy). Cell viability was measured 8 days after radiation and normalized to nonirradiated cells. Two-way ANOVA with Dunnett’s multiple-comparison test. ***P < 0.001. Blue indicates the *P* value when comparing NT-sh and Syx-sh1; red indicates the *P* value when comparing NT-sh and Syx-sh2. (**G**) Kaplan-Meier survival curves of mice orthotopically transplanted with GBM12 cells transduced with indicated shRNAs. *n* = 8–10 mice per group.

The chemotherapeutic agent TMZ improves patient overall survival and is part of the standard of care for GBM, along with surgery and radiation therapy (39). TMZ treatment generates DNA lesions, leading to replication fork collapse, G2/M cell cycle arrest, and cell apoptosis, and tumor cells with low or no expression of MGMT are particularly sensitive to this agent. Since silencing of either *Syx* or *Dia1* expression increased DNA damage in GBM cells, we postulated that targeting Syx-Dia1 signaling might cooperate with TMZ in suppressing cell growth. To test this hypothesis, we transduced U251 cells with increasing amounts of *Syx*-sh carrying lentiviruses, in order to achieve increasing levels of *Syx* depletion (Figure 7A). After infection, cells were treated with different concentrations (10–300 μM) of TMZ, and cell viability was then assessed using the Cyquant assay (Figure 7B).

As expected, knockdown of Syx reduced cell growth (Figure 7, B and C). TMZ also resulted in a dose-dependent decrease of cell growth in U251 cells, which are sensitive to TMZ due to MGMT promoter methylation (Figure 7, B and C). Importantly, the combination of Syx depletion with TMZ resulted in a profound and dose-dependent loss of cell viability (Figure 7, B and C). We then tested TMZ synergy with Syx targeting and observed a Combination Index (CI) of < 1, suggestive of synergistic interaction between the 2 across all 3 × 4 dose matrix tested (Figure 7C). Since both TMZ and Syx depletion cause accumulation of cells in G2/M and DNA damage (3) (Figure 2A; Figure 3, C and D; and Supplemental Figure 3, A–D), we further assessed the effect of this combination strategy on cell cycle progression, mitotic entry, and DNA DSBs. The combined treatment increased both the extent of G2/M arrest (at low concentrations of TMZ) and levels of DSBs (detected by γH2AX), and it decreased mitotic entry (detected by pHH3) (Figure 7, D and E). However, TMZ treatment did not further decrease cyclin levels or increase the level of cleaved caspase 3 induced by *Syx* knockdown (Supplemental Figure 7A), suggesting that they act by nonoverlapping mechanisms.

As depletion of Syx inhibited the growth of both TMZ-sensitive (GBM12) and -resistant (GBM10, GBM14) lines (Figure 1C and Supplemental Figure 1), we postulated that targeting Syx could synergize with TMZ even in lines with acquired TMZ resistance. To test this hypothesis, we utilized a previously established U251 subclonal line selected for TMZ resistance (U251TMZ) (40). Indeed, we verified that the half-maximal inhibitory concentration (IC_50_) of TMZ for U251TMZ is approximately 10-fold higher than its parental U251 line (58.2 ± 5.1 μM versus 4.3 ± 1.4 μM, respectively; mean ± SEM) when assayed by the Cyquant method after TMZ exposure. Similar to parental U251 cells, the combination of Syx depletion with TMZ resulted in a profound, dose-dependent, and synergistic loss of viability in U251TMZ cells (Supplemental Figure 7, B and C). However, TMZ treatment did not further potentiate the effect of Syx knockdown in inducing DSBs or suppressing mitosis entry (Supplemental Figure 7D). A similar decrease in cell viability was also observed in endogenously TMZ-resistant T98G cells, where the combination of Syx depletion plus TMZ increased DSBs at higher TMZ levels (30 μM and 100 μM) (Supplemental Figure 7, E and F). Overall, these data indicate that targeting the Syx-Dia1 signaling pathway inhibits GBM growth and cooperates with TMZ to induce GBM cell death.

Ionizing radiation is the other arm of the standard of care for GBM. Radiation therapy induces DNA DSBs, leading to cancer cell death. To determine whether targeting Syx signaling can potentiate cell response to RT, we tested the combined effect of Syx depletion and radiation therapy in U251 and T98G cells. In both cell lines, RT caused a dose-dependent decrease of cell viability, which was significantly increased by concomitant depletion of endogenous Syx (Figure 7F and Supplemental Figure 7G). In agreement with this, the median survival of mice orthotopically implanted with Syx-knockdown GBM12 cells and treated with radiation for 5 days (2 Gy × 5 fractions) was 94 days, compared with 57.5 days for the NT-sh/RT control (Figure 7G). The data argue that targeting Syx sensitizes GBM cells to radiation therapy.

Finally, recent studies reported the induction of the endoplasmic reticulum unfolded protein response (UPR^ER^) by DDR (41) as well as the inhibition of UPR^ER^ by YAP signaling (42). Interestingly, Syx depletion promoted the UPR^ER^, as evidenced by the upregulation of key markers including BiP, IRE1α, CHOP, and JNK phosphorylation (Supplemental Figure 7H). The UPR^ER^ cascade can often lead to autophagy for the maintenance of organelle and cellular homeostasis. We observed an increase in LC3 lipidation (LC3-II), indicating the initiation of autophagy by Syx depletion, but no decrease in the lysosomal marker p62/SQSTM1 (Supplemental Figure 7H).

## Discussion

**Figure 8:**
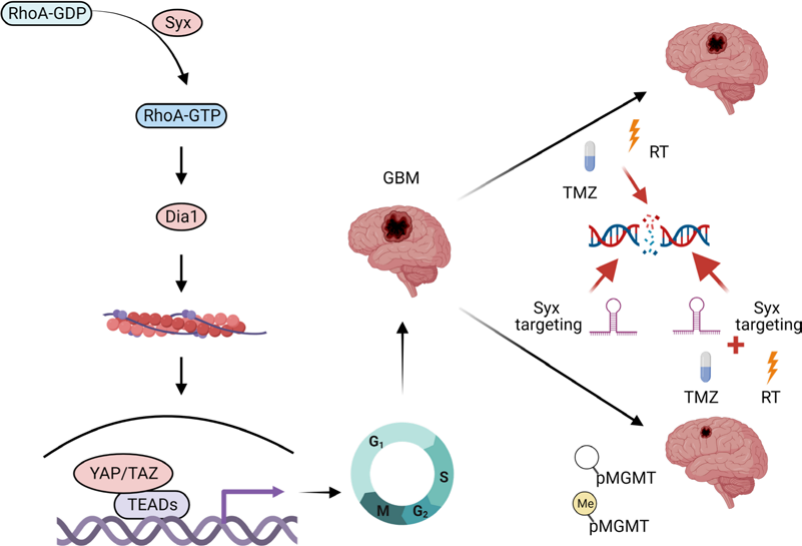
Schematic diagram of the Syx-RhoA-Dia1-YAP/TAZ signaling axis in cell cycle progression, DNA damage, and therapy resistance in GBM. The RhoA GEF Syx activates RhoA and its downstream effector Dia1. This results in increased YAP/TAZ stability, nuclear translocation, and transactivation activity. The pathway contributes to cell cycle gene regulation, promoting GBM cell cycle progression and increased tumor growth. Syx targeting, like TMZ and radiation therapy (RT), promotes DNA double-strand breaks and potentiates GBM response to these treatments. This approach is independent of the MGMT promoter methylation status, presenting a potential therapeutic strategy for GBM.

Despite multimodal therapy with surgery followed by radiation and TMZ, GBM is a universally lethal disease. Aggressive growth and dissemination of tumor cells in brain parenchyma are major factors contributing to this dismal outcome. The “go or grow” hypothesis argues that cancer cells either migrate or proliferate, and based on this, they exhibit different sensitivities to therapies that selectively target migrating or proliferating cells. Therefore, targeting both glioma cell migration and growth may be essential for optimal management of GBM (43). We previously reported that the Syx-RhoA signaling axis is active in glioma cells and that suppressing Syx action blocks both random and directed glioma cell migration by reorganizing the cytoskeleton and disrupting microtubule bundling and capture at leading cell edges (8). Here, we show that activation of Syx also results in alterations of cell cycle regulators at the mRNA levels, promoting cell cycle progression and GBM tumor growth. These effects are promoted by the Syx-mediated activation of RhoA, by selective activation of the downstream effector Dia1, and,at least in part, by YAP/TAZ signaling. Importantly, targeting this pathway cooperates with both RT and TMZ, the standard of care for GBM (Figure 8). Combined, these data point to a key role for Syx in both GBM growth and migration and suggest that this pathway can be exploited for GBM therapy.

Upregulation of RhoA expression and activation results in increased cell migration, invasion, and tumor growth (5). In agreement, our previous work has demonstrated that Syx is part of the Crumbs polarity complex and promotes directed glioma cell migration via its downstream effectors RhoA and Dia1 (7, 8). Increased expression/activation of YAP/TAZ promotes tumorigenesis and correlates with poor clinical outcome in patients with GBM (44–47). Here, we identified a Syx-RhoA-Dia1 signaling axis affecting transcriptional levels of multiple cell cycle regulators, at least in part via YAP/TAZ nuclear signaling, to promote glioma cell growth. The mechanism by which Syx-mediated activation of RhoA and Dia1 regulates phosphorylation, nuclear localization, and activation of YAP/TAZ signaling is unclear. It is plausible that both Hippo-dependent and -independent components are involved in this newly identified signaling axis. YAP1-S127 and S381 residues (and equivalent TAZ sites) are the most relevant phosphorylation sites by the Hippo pathway kinase LATS (48). Consistent with the function of these sites, depletion of Syx increased S127 and S381 phosphorylation, increased cytoplasmic retention, and promoted proteasomal degradation of YAP/TAZ (Figure 6, A and B, and Supplemental Figure 5, C and D). Moreover, ectopic expression of a constitutive active YAP1 mutant (YAP1-S127/381A) was able to at least partially suppress DSBs induced by Syx knockdown (Supplemental Figure 5E). Hippo-independent mechanisms may involve phosphorylation of YAP1-S127 and S381 by kinases other than LATS (14), a hypothesis supported by the less prominent role of Hippo signaling in cytoskeleton-mediated YAP/TAZ modulation (20, 38). Finally, the ability of Syx and YAP/TAZ to associate with 14-3-3 family members and Amot (9, 11, 12) provides another unexplored mechanism of potential cross-regulation.

Notably, YAP/TAZ-independent mechanisms may also contribute to the Syx-mediated promotion of GBM cell growth. Indeed, Syx depletion decreased both basal and serum-induced SRF/MRTF-A activity, and treatment of GBM cells with CCG-203971, a specific inhibitor of SRF/MRTF signaling, decreased GBM cell growth (Supplemental Figure 6) (47). Taken together, the data argue for the development of Syx-specific inhibitors. Syx-KO mice develop normally (7), increasing the possibility that Syx-targeted therapeutics with tolerable toxicity profiles can be identified for either systemic or local administration in patients with cancer.

Accumulation of DNA damage by multiple mechanisms (radiation therapy, chemotherapy, genetic mutations) triggers DDR, which surveils DNA integrity and induces cell cycle checkpoints and DNA repair mechanisms. Prolonged mitosis is known to cause structural aberrations of chromosomes and DNA breaks (49–51). Our data show that glioma cells depleted of endogenous Syx are arrested at G2/M, while a fraction of them undergo a prolonged mitosis (Figure 2D). We postulate that the fraction of cells undergoing mitosis is responsible for the increase in DNA damage, a hypothesis we did not test directly. Both Survivin and Cdc20 are key proteins that ensure proper chromosome alignment and segregation during mitosis (23, 24). Thus, it is plausible that the prolonged mitosis and the increase in DNA DSBs observed in Syx-depleted cells are caused by downregulation of Survivin and Cdc20.

We postulated that the defects in cell division and the resulting DDR induced by targeting the Syx signaling pathway can be exploited for GBM therapy, as they can ultimately lead to cell apoptosis. In support of this hypothesis, combination of Syx depletion and TMZ synergistically inhibited cell growth in both TMZ-sensitive and TMZ-resistant cell lines (Figure 7, A–C, and Supplemental Figure 7, B and C). While the exact mechanisms by which Syx depletion and TMZ cooperate to promote DNA damage and inhibit GBM cell growth (Figure 7, A–E, and Supplemental Figure 7, A–F) are not yet defined, the data suggest a potential role for Syx in modulating cellular responses to TMZ. Since both TMZ and radiation therapy induce DDR, we postulated that depletion of Syx may sensitize cells to RT, a hypothesis that was validated in vitro in TMZ-sensitive (U251, Figure 7F) and TMZ-resistant (T98G, Supplemental Figure 7G) cells and supported in vivo in GBM12 orthotopic xenografts (Figure 7G). Future studies will focus on targeting Syx signaling in already-established GBM tumors by using inducible shRNAs, more accurately reflecting the clinical course of the disease.

One concern is that p53 deregulation, which frequently occurs in GBM (52), can lead to defects in apoptosis upon induction of the DDR. However, we observed similar growth defects by targeting Syx-Dia1-YAP/TAZ signaling in either TP53-mutant (U251, LN229, T98G, and GBM12) or TP53-WT (GBM10, GBM14) cell lines. These data argue that Syx-directed therapeutics could target GBM cells irrespective of their p53 status. We examined the role of Syx signaling in UPR^ER^ and autophagy, 2 interconnected pathways that can be regulated by both the DDR and YAP signaling and can lead to apoptosis in the absence of p53, if cellular homeostasis cannot be maintained (53, 54). Syx depletion promoted the UPR^ER^, induced proapoptotic markers CHOP and JNK, and initiated but failed to complete autophagy in our cells, suggesting a loss of cellular homeostasis (Supplemental Figure 7H). Nonetheless, a recent study reported increased production of autolysosomes, fusion products of autophagosomes and lysosomes, in GBM cells upon Syx KO (55). Therefore, while the mechanistic details are still unclear, a potential role for Syx in regulating UPR^ER^, autophagy, and cellular metabolism exists and requires further examination.

In summary, we provide evidence implicating the Syx-Dia1-YAP1 signaling pathway in GBM tumorigenesis and uncover its role in cell cycle progression and cell growth. Although we only explored the role of the potentially novel Syx-Dia1-YAP1 signaling axis in GBM, it is plausible that this signaling pathway is also involved in other cancer types, such as a subset of breast, ovarian, and pancreatic adenocarcinoma that have been shown to exhibit *Plekhg5* (Syx) amplification (cBioPortal) (56). Moreover, the Hippo-YAP/TAZ signaling pathway is one of the 10 canonical cancer pathways identified in the pancancer exploration by The Cancer Genome Atlas (TCGA) (57). Therefore, the role of Syx signaling in Hippo-YAP/TAZ–activated tumors such as many non-CNS tumors (e.g., mesothelioma, lung cancers; ref. 58) and CNS tumors (e.g., chordoma and ependymoma; refs. 59, 60) is worth exploring in the future. Combined with its ability to promote directed cell migration, the Syx signaling pathway presents a unique target for GBM therapy. This is further underscored by evidence that targeting Syx in combination with TMZ or RT, the current standard therapy, strongly suppresses GBM cell growth in vitro. The clinical translation of these findings is limited by the lack of specific Syx or Dia1 small molecule inhibitors and the poor blood brain barrier penetration of available YAP targeting agents like verteporfin. However, other exchange factors have been targeted, suggesting that specific inhibitors of the Syx-RhoA interaction can be generated (61, 62). We postulate that targeting Syx, as the most upstream pathway member, will provide maximal efficacy. However, it is possible that other RhoGEFs can also selectively activate RhoA-Dia1-YAP/TAZ signaling in GBM cells. Understanding the nodes of vulnerability to this pathway in different patient tumors will be essential for the development of optimal targeted therapies for this deadly disease.

## Methods

### Study design.

Experiments were designed to determine the role of Syx in GBM cell growth, to identify underlying molecular mechanisms of action, and to assess whether targeting Syx signaling can increase GBM sensitivity to chemotherapy and radiation therapy. Two independent shRNAs for each target gene (Syx, Dia1, and YAP1) and multiple GBM cell lines (3 conventional and 3 PDX lines) were used in the study, and cells were maintained for short time periods (approximate 3–4 weeks) to prevent genetic drifting. Cell growth and qPCR assays were carried out with multiple replicates (indicated in figure legends) with 3 technical repeats. For time-lapse fluorescence imaging experiments in living cells, limited amount of light was used to excite fluorescently labeled proteins to prevent phototoxicity. The 2 survival studies in vivo were performed in 5 animals per group and 8–10 animals per group, respectively, and this provided sufficient power to assess statistical significance when all animals were included for the determination of survival difference between different groups.

### Constructs, reagents, and antibodies.

The Hs-Syx and pHIV-H2BmRFP (RFP-H2B) expression plasmids were obtained from GenScript (Ohu22775C, NM_198681 transcript variant 2 mRNA ORF clone) and Addgene (plasmid 18982), respectively. The pEYFP-Dia1(ΔN3) (CA-Dia1) and pSinLuc constructs were gifts from S. Narumiya (Kyoto University, Kyoto, Japan) (63) and Yasuhiro Ikeda (Mayo Clinic, Rochester) (64), respectively. The pEYFP-Dia1(ΔN3)-Syx(C) construct (chimera) was generated previously (8). The YAP1-S127/381A (YAP1-AA) was generated using the p2xFLAGhYAP1-S127A (Addgene, 17790; ref. 65) backbone and Y381A QuickChange primers (5′-tatcactctcgagatgagGCtacagacagtggactaagc-3′ and 5′-gcttagtccactgtctgtaGCctcatctcgagagtgata-3′). pcDNA3.1(+)-myc was used as the transfection control for Figure 6E and Supplemental Figure 5E. Luciferase plasmids, including 8xGTIIC-luciferase (Addgene, plasmid 34615; ref. 38), SRF-RE luciferase (pGL4.34[luc2P/SRF-RE/Hygro], Promega E1350), and human Renilla luciferase (pGL4.74[hRluc/TK], Promega, E6921) were used in the study. The MISSION shRNAs in the pLKO.1 lentiviral vector with a puromycin resistance gene were from MilliporeSigma. Product identification numbers for each shRNA are listed — NT-sh, SHC002; Syx-sh1, TRCN0000130291; Syx-sh2, TRCN0000128190; Dia1-sh1, NM_0052192.2-2523s1c1; Dia1-sh2, NM_005219.2-2557s1c1; YAP1-sh1, NM_006106.2-1232s1c1; Yand AP1-sh2, NM_006106.2-1373s1c1.

Chemicals included TMZ (MilliporeSigma, T2577), RO-3306 (MilliporeSigma, SML0569), CCG-203971 (Tocris Bioscience, 5277), and MG132 (MilliporeSigma, C2211). Antibodies used for immunoblotting and immunofluorescence included: Syx (Abnova, H00057449-M01, clone 5A9), GAPDH (Cell Signaling Technology, 2118), α-tubulin (Sigma, T5168), pS10-HH3 (Cell Signaling Technology, 9701), HH3 (Cell Signaling Technology, 4499), Cdc20 (Cell Signaling Technology, 4823), Survivin (Cell Signaling Technology, 2808), PARP (Cell Signaling Technology, 9542), cCas3 (Cell Signaling Technology, 9661), pS139-H2AX (γH2AX, Cell Signaling Technology, 2577), H2AX (Cell Signaling Technology, 7631), p27 (Santa Cruz Biotechnology, sc528), p21 (Cell Signaling Technology, 2946), Cyclin D1 (Abcam, ab134175; or Cell Signaling Technology, 2978), Cyclin E2 (Cell Signaling Technology, 4132), Cyclin A2 (Abcam, ab38), Cyclin B1 (Cell Signaling Technology, 4138), Dia1 (BD, 610848), YAP1/TAZ (Santa Cruz Biotechnology, sc-101199), YAP1 (Cell Signaling Technology, 14074), TAZ (BD, 560235), pS127YAP/pS89TAZ (Cell Signaling Technology, 4911), pS397YAP1 (corresponding to pS381 of YAP2, Cell Signaling Technology, 13619), BiP/GRP78 (Cell Signaling Technology, 3177), IRE1α (Cell Signaling Technology, 3294), CHOP (Cell Signaling Technology, 2895), pT183/Y185-JNK (Cell Signaling Technology, 9251S), JNK (Santa Cruz Biotechnology, sc-474), LC3-I/II (Acris, AM20212PU-N), and p62/SQSTM1 (Cell Signaling Technology, 5114S).

### Cell culture, transfection and transduction.

GBM conventional cell lines (U251, LN229, T98G), PDX lines (GBM10, GBM12, GBM14), and the U251TMZ resistance line (U251TMZ) (40) were maintained in DMEM (Corning, 10-017-CV) supplemented with 10% FBS (Thermo Fisher Scientific, 10437028), 2 mM L-glutamine (Corning, 25005CI), and 1% nonessential amino acids (Corning, 25055CI). Penicillin and streptomycin (Corning, 30002CI) were included in the PDX culture media. All PDX lines used in this study, U251, U251TMZ, and T98G lines were provided by Jann Sarkaria (Mayo Clinic, Rochester). LN229 were purchased from ATCC (CRL-2611). Conventional and PDX lines were maintained in culture for fewer than 15 and 5 passages, respectively. Transfection was performed using Lipofectamine 200 (Invitrogen) according to the manufacturer’s instructions. RNA silencing in this study was described previously using the MISSION shRNA Lentiviral Transduction (Sigma-Aldrich) (8). Infected cells were selected with puromycin (2.5 μg/mL for U251, LN229, GBM12, GBM14, and 3.3 μg/mL for GBM10) for 48 hours prior to experiments. The amount of Syx shRNA–expressing viruses varied for experiments involving TMZ. For mitotic live-cell imaging, U251 cells expressing RFP-H2B were transduced with lentiviral shRNAs, followed by puromycin selection as above.

### Animals and orthotopic injections.

To generate GBM xenografts, 4- to 5-week-old female athymic nude (Hsd: Athymic Nude-Foxn1^nu^, order code 069) (Harlan) were anesthetized with isoflurane, and short-term explant cultures of GBM12 cells transduced with the luciferase-expression (pSinLuc) and NT or Syx shRNAs lentiviruses were implanted into the brain through intracranial injection, as described previously (66). Tumor cells (3 × 10^5^ in 5 μL per mouse) were implanted 2 mm lateral and 1 mm anterior to bregma, at 3 mm depth. Animals were monitored daily and maintained until reaching a moribund state. Tumor growth was monitored once a week. Briefly, mice were injected i.p. with luciferin (150 mg/kg/0.1 mL), anesthetized with isoflurane, and imaged with the IVIS Spectrum (Caliper Life Sciences) 10–15 minutes after injection. Moribund mice were deeply anesthetized by i.p. injection of 90 mg/kg pentobarbital and euthanized by transcardial perfusion of PBS followed by 4% paraformaldehyde. Brains from mice were resected, cut into 4 coronal sections of equivalent thickness, and fixed in 4% paraformaldehyde overnight at 4°C. Subsequent formalin fixation and paraffin-embedding (FFPE) was performed, and 5 μm–thick tissue sections were used for the RNAscope assay. For the dosing study, GBM12 cells expressing NT-sh or Syx-sh2 shRNA lentiviruses (6.5 × 10^4^ in 3 μL per mice) were orthotopically injected into the brain. These mice (female, 7- to 10-week-old, NCI athymic NCr-nu/nu, strain code 553) were then randomized and treated 4 days after implantation. Animals were dosed with radiation therapy (2 Gy fractions for 5 days) or sham.

### Immunoblotting.

Cells were lysed with RIPA buffer (50 mM Tris [pH 7.4], 150 mM NaCl, 1% NP-40, 0.5 % deoxycholic acid, 0.1% SDS) supplemented with protease inhibitors (RPI, cocktail III) and phosphatase inhibitors (Pierce). In some cases, cells were lysed with 2× Laemmli Sample Buffer (LSB) followed by homogenization through a 29-gauge needle. Protein quantification was assessed using the BCA Protein Assay Kit (Pierce) or the RC DC Protein Assay (Bio-Rad). Immunoblotting was performed according to standard protocols with ECL (GE Healthcare) reagents. For the synchronization experiment, U251 cells were treated with RO-3306 (9 μM) for 20 hours. Cells at different time points after treatment were collected and lysed in 2× LSB.

### RNAScope.

The RNAscope 2.5 HD Brown assay (Advanced Cell Diagnostics) was used to detect mRNAs in 5 μm mouse tissue sections. In situ hybridization was performed according to the manufacturer’s protocol with the Hs-Plekhg5 probe (NM_001042663.1, target region 689–1,750, catalog 415321). Images were captured using an AT2 slide scanner and ImageScope software (Leica Biosystems).

### Cell cycle analysis.

Cell cycle progression was determined by flow cytometry using propidium iodine to label DNA content. Briefly, cells at subconfluency were trypsinized, washed twice with PBS, and fixed in ice-cold 70% ethanol at –20°C overnight. Fixed cells were then incubated with 1 mg/mL RNase A in 0.1% sodium citrate at 37°C for 15 minutes, and DNA was stained with 100 μg/mL propidium iodide at room temperature for 15 minutes prior to flow cytometry. DNA content was detected with the Accuri C6 (BD Biosciences) or the Life Attune NxT cytometers (Thermo Fisher Scientific). FlowJo and FCS express 5 were used for data analysis. For the combined Syx targeting and TMZ experiment, shRNA lentivirus–infected cells were treated with different concentrations of TMZ for 3 days.

### Cell growth.

Cell growth was assessed using the MTT (MilliporeSigma) or Cyquant (Thermo Fisher Scientific) assays following manufacturer protocols. The Cyquant assay was used for experiments involving TMZ. For the MTT assay, cells were plated (U251, 4,000 cells; GBM10, 5,000 cells; GBM12, 5,000 cells; GBM14, 3,000 cells per well) in triplicate in 96-well plates and allowed to grow for 5–8 days. For the Cyquant assay, U251 cells were plated (500 cells per well) and allowed to grow for 24 hours prior to 5- to 6-day treatment with indicated concentrations of TMZ. For the radiation experiments, U251 and T98G cells expressing shRNAs were plated (4,000 cells per well), subjected to radiation a day later using a RAD-160 Biological Irradiator (Precision X-Ray), and cell viability was assessed by the MTT assay 8 days after radiation. Synergy analysis was performed using the Calcusyn software (Biosoft) with the Chou-Talalay method. CI was used to describe synergistic (CI < 1), antagonistic (CI > 1), or additive (CI = 1) drug interactions. Heatmaps were generated using the GraphPad PRISM software.

### qPCR.

Total RNA was isolated using Trizol (Invitrogen) followed by PureLink RNA minikit (Ambion) according to manufacturer’s protocol. The high-capacity cDNA reverse transcriptase kit (Applied Biosystems) was used to convert RNA to cDNA. qPCR reactions were carried out using the TaqMan FAST Universal PCR master mix (Applied Biosystem), in a ViiA 7 or 7900 HT Real-Time PCR system (Applied Biosystems). Data analysis was performed using the RQ Manager (Applied Biosystem), and data were normalized to GAPDH or β-actin. The assay IDs for TaqMan Gene Expression Assay are: GAPDH (Hs99999905_m1), ACTB1 (Hs99999903_m1), Plekhg5 (Syx, Hs00299154_m1), DIAPH1 (Dia1, Hs00946556_m1), CTGF (Hs00170014_m1), BIRC5 (Survivin, Hs00153353_m1), CDKN1A (p21, Hs00355782_m1), CDKN1B (p27, Hs00153277_m1), Cdc20 (Hs00426680_mH), CCNA2 (Hs00996788_m1), CCNB1 (Hs01030103_m1), CCNE2 (Hs00180319_m1), and YAP1 (Hs00902712_g1).

### Immunofluorescence and live-cell imaging.

Cells grown on coverslips were fixed in 4% paraformaldehyde/0.12M sucrose/PBS solution at room temperature for 15 minutes, permeabilized with 0.2% Triton X-100/PBS for 5 minutes, and blocked with Protein-Block reagent (Dako, X090930-2) at room temperature for 30 minutes. Proteins were then stained with primary antibodies overnight at 4°C and then Alexa Fluor–conjugated secondary antibodies (Invitrogen) for 1 hour at room temperature. Antibodies were diluted in antibody diluent (Dako, S302281-2). Nuclei were visualized by DAPI (MilliporeSigma) staining. Cells were mounted with Aqua Poly/Mount (Polysciences) and imaged using a Zeiss LSM510 META or LSM880 laser confocal microscope under a 40× objective. Z-series of images were acquired, and maximum intensity projection images were generated before data analysis and are shown in figures. γH2AX foci and YAP/TAZ subcellular localization were manually analyzed using ImageJ (NIH). For live-cell imaging, images were captured using an Olympus IX83 imaging system equipped with a Stage Top Incubator (Tokai Hit). To examine cell apoptosis in living cells, U251 cells were treated with 4 μM CellEvent Caspase-3/7 green detection reagent (Molecular Probes) prior to imaging. U251 cells expressing RFP-H2B and NT- or *Syx*-sh were plated the day before imaging. Images were acquired every 10 or 20 minutes over a 24-hour period with a 20× phase objective. Phase-contrast images and RFP-H2B signals were used to identify cells undergoing mitosis. Initiation of mitosis was identified when cells started to shrink and round up, and completion of mitosis was identified when 2 daughter cells were separated. The H2B videos were generated using ImageJ.

### Luciferase reporter assay.

U251 and T98G cells were plated and transfected the next day with the 8xGTIIC-luciferase and pGL4.74[hRluc/TK] plasmids. The pGL4.74[hRluc/TK] Renilla luciferase construct was used to normalize for transfection efficiency. Medium was replaced 4 hours after transfection. Cells were lysed 24 hours after transfection, and the lysates were subjected to dual-luciferase reporter (DLR) assay (Promega) according to the manufacturer’s protocol. Luciferase was detected with a Veritas Microplate Luminometer (Turner Biosystems). To assess MRTF-SRF transcriptional activity, U251 cells were transfected with the SRF-RE luciferase and the human Renilla luciferase plasmids. For serum starvation, cells were replated the next day and incubated in serum-free media for 18 hours. Serum stimulation was performed by adding 20% serum to serum-starved cells and incubating for 6 hours at 37°C.

### Statistics.

Statistical analysis was performed using the GraphPad Prism software. For in vitro experiments, 1- or 2-way ANOVA with Dunnett’s multiple-comparison test was used to determine statistical differences between 2 experimental groups. For shRNA experiments, statistical comparison between each shRNA of Syx, Dia1, or YAP1 and NT-sh control was performed. Bar graphs present the data (mean ± SD or mean ± SEM) from multiple experiments or data points. For the animal survival experiment, the Kaplan-Meier method with the log-rank test was used to compare the survival difference between each Syx shRNA group and the NT-sh control. Experimental details are indicated in the figure legends. *P* < 0.05 was considered statistically significant.

### Study approval.

Animals were treated and care-monitored in adherence to the protocol approved by the Mayo Clinic IACUC.

### Data availability.

All data are available in the main text or the supplementary materials.

## Author contributions

Conception and design were contributed by WHL and PZA; development of methodology was contributed by WHL, LJLT, and JNS; data acquisition was contributed by WHL, RWF, LMC, LJLT, and JC; analysis and interpretation of data were contributed by WHL and PZA; and manuscript preparation was contributed by WHL and PZA.

## Supplementary Material

Supplemental data

Supplemental video 1

Supplemental video 2

Supplemental video 3

Supporting data values

## Version 1 07/10/2023

Electronic publication
