# Supplementary material for: A Syx-RhoA-Dia1 signaling axis regulates cell cycle progression, DNA damage, and therapy resistance in glioblastoma
Source: JCI Insight. 2023 Jul 10;8(13):e157491. doi: 10.1172/jci.insight.157491 (PMC10371349; doi:10.1172/jci.insight.157491)
Supplement: Supplemental data [file jciinsight-8-157491-s216.pdf]

A Syx-RhoA-Dia1 signaling axis regulates cell cycle progression, DNA damage, and therapy resistance in glioblastoma

One Sentence Summary: Syx promotes growth and therapy resistance in glioblastoma.

Authors: Wan-Hsin Lin[1], Ryan W. Feathers[1], Lisa M. Cooper[1], Laura J. Lewis-Tuffin[1], Jiaxiang Chen[1], Jann N. Sarkaria[2], and Panos Z. Anastasiadis[1]*

Affiliations:

[1]Department of Cancer Biology, Mayo Clinic, 4500 San Pablo Road, Jacksonville, FL 32224, USA. Phone: 904-953-6005

[2]Department of Radiation Oncology, Mayo Clinic, Rochester, MN 55905, USA.

*Corresponding author. Email: anastasiadis.panos@mayo.edu

Supplemental Videos

Video 1. U251 cells transduced with RFP-H2B and NT-sh viruses. Images were acquired every 10 min for 24 hr. Play rate, 10 frames per second.

Video 2. U251 cells transduced with RFP-H2B and Syx-sh1 viruses. Images were acquired every 10 min for 24 hr. Play rate, 10 frames per second.

Video 3. U251 cells transduced with RFP-H2B and Syx-sh2 viruses. Images were acquired every 10 min for 24 hr. Play rate, 10 frames per second.

Supplemental Figure 1

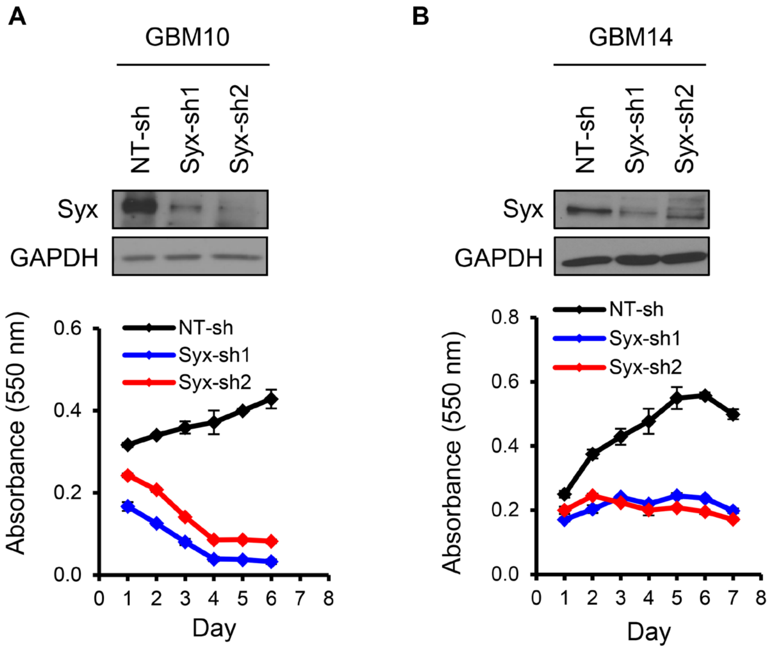

Supplemental Figure 1. Syx knockdown decreases cell growth in GBM PDX lines. (**A** and **B**) Immunoblot analysis of Syx and GAPDH in lysates from GBM10 (**A**) and GBM14 (**B**) cells transduced with indicated shRNAs (top). Cell viability over indicated time for each cell population was measured by the MTT cell proliferation assay (bottom). Shown are representative graphs with three technical replicates of 3-4 biological replicates. Graphs represent the mean ± SD.

Supplemental Figure 2

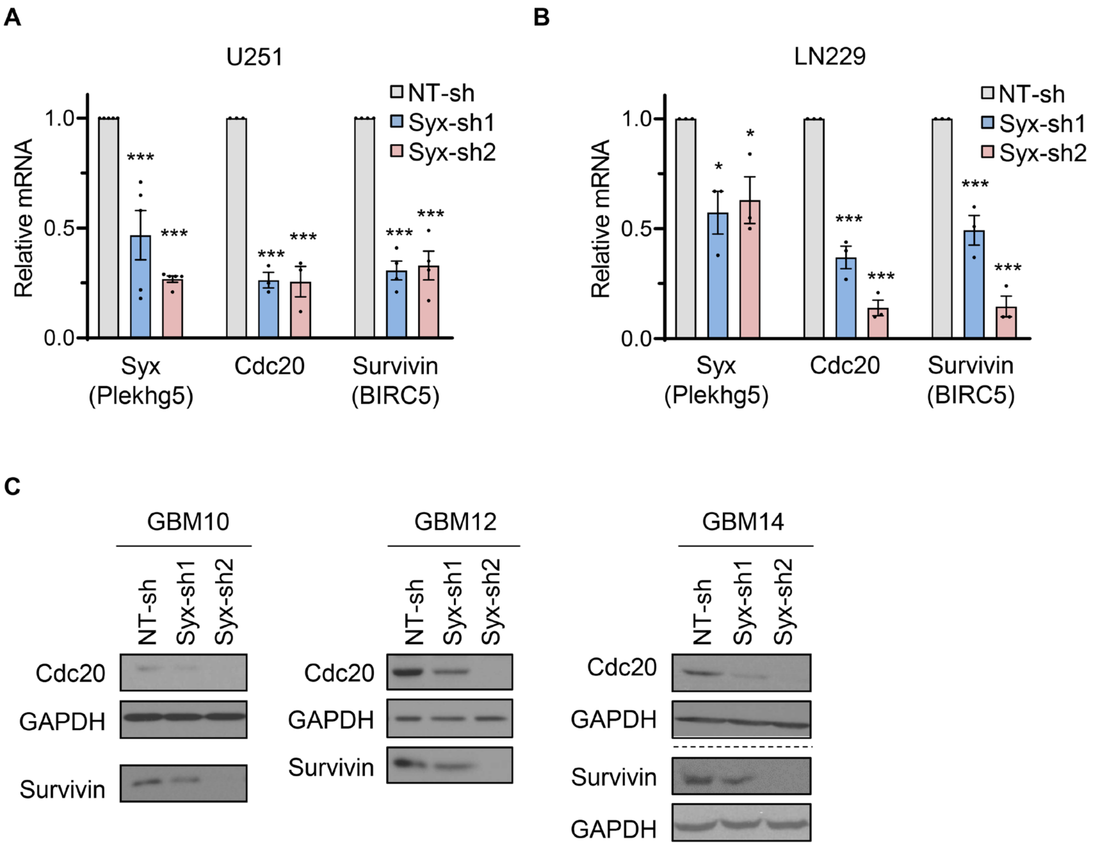

Supplemental Figure 2. Syx depletion downregulates expression of mitosis regulators. (**A-C**) RT-qPCR (**A**, **B**) and immunoblot (**C**) analysis of the expression of mitosis regulators Cdc20 and Survivin (BIRC5) as well as Syx (Plekhg5) in U251 (**A**), LN229 (**B**) cells and three PDX lines (**C**, GBM10: left, GBM12: middle, GBM14: right) that are grown at sub-confluency and expressing indicated shRNAs. Different biological samples are separated by dashed lines. Same biological samples run at different times are indicated by larger white space. GBM12 samples were run in parallel. Graphs represent the mean ± SEM of 3-5 biological replicates of relative mRNA expression of indicated genes normalized by GAPDH. One-way ANOVA with Dunnett's multiple comparisons test, $*P < 0.05$, $***P < 0.001$.

Supplemental Figure 3

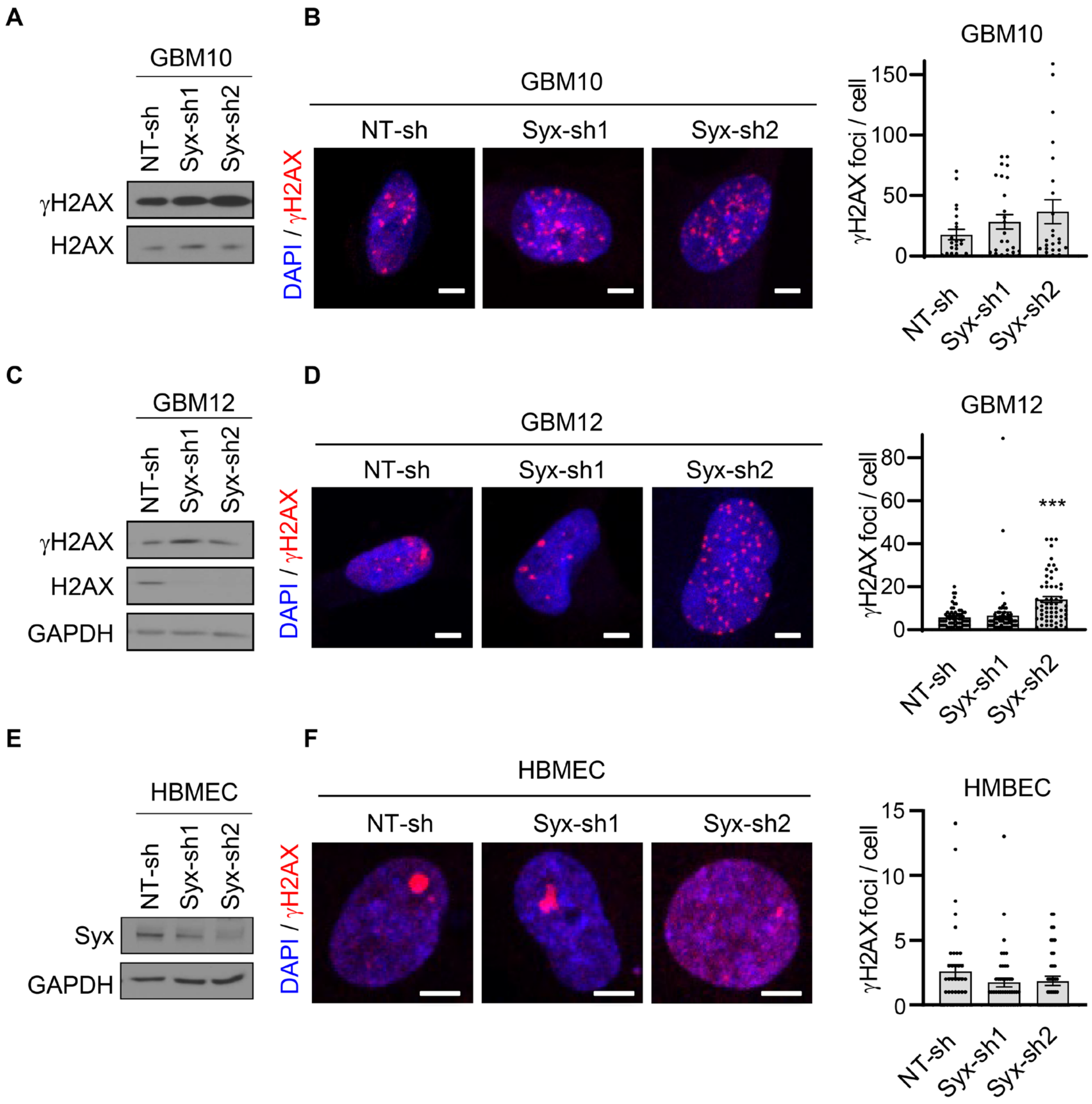

Supplemental Figure 3. Syx knockdown induces DNA damage in GBM PDX lines but not in brain endothelial cells. (**A**-**F**) Immunoblots (**A**, **C**, **E**) and immunofluorescence staining (**B**, **D**, **F**) of phosphorylated H2AX at Ser-139 (γH2AX) in GBM10 (**A** and **B**), GBM12 (**C** and **D**) and human brain endothelial (HBMEC, **E** and **F**) cells. Same samples with equal loading amount were run in parallel (**A** and **C**). GAPDH for panel **A**, see GBM10 in Supplemental Figure **2C**. Images (**B**, **D**, **F**) show staining for γH2AX (red) and nucleus (DAPI). Scale bar, 5 μm. Shown are maximum intensity projection images. Graphs (**B**, **D**, **F**) on the right represent the average ± SEM number of γH2AX foci per cell. GBM10, n = 22-26; GBM12, n = 61-62; HBMEC, n = 38-45. One-way ANOVA with Dunnett's multiple comparisons test, $^{***}P < 0.001$.

Supplemental Figure 4

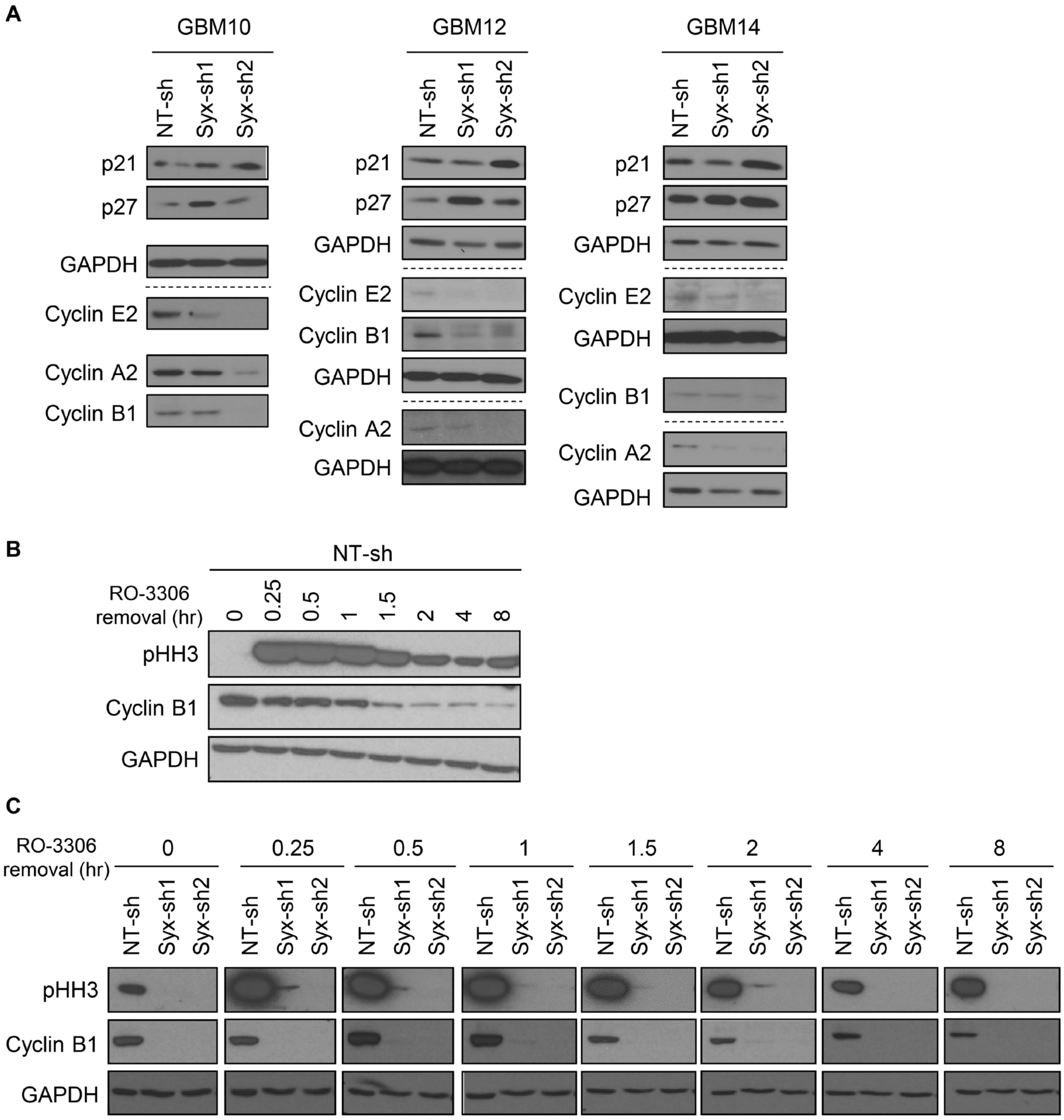

Supplemental Figure 4. Syx knockdown alters expression of cell cycle regulators. (**A**) Immunoblot analysis of p21, p27, Cyclin E2, Cyclin A2, Cyclin B1 and GAPDH in GBM PDX lines. GBM10, left; GBM12, middle; GBM14, right. (**B** and **C**) Immunoblot analysis of the levels of phosphorylated histone H3 at Ser10 (pHH3), Cyclin B1 and GAPDH in U251 cells before (0 hr) and after the removal of the G2 phase block RO-3306 (9 μM) at indicated time points. The same lysates from NT-sh (**B**) are also loaded side by side to the Syx knockdown lysates (**C**) for comparison. Different biological samples are separated by dashed lines. Same biological samples run at different times are indicated by larger white space. For GBM10, see GAPDH loading control in Supplemental Figure **2C** for Cyclins. All other blots were run in parallel.

Supplemental Figure 5

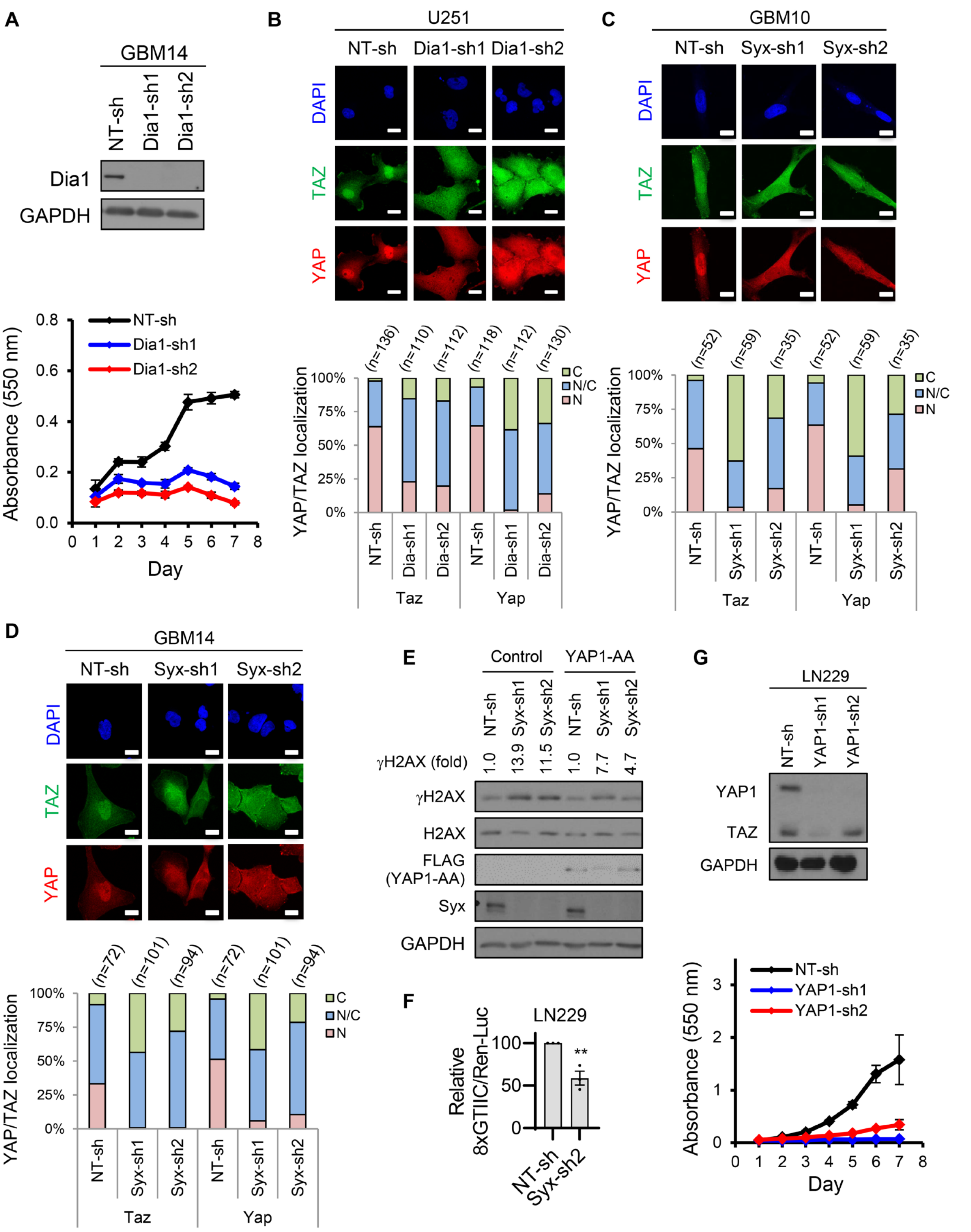

Supplemental Figure 5. Downregulation of the Syx-Dia1 signaling axis reduces cell growth and increases cytoplasmic retention of YAP/TAZ. (**A**) Immunoblot analysis of Dia1 and GAPDH in lysates from GBM14 cells transduced with indicated shRNAs (top). Graph shown is representative of 2 biological repeats and presents cell viability (mean ± SD) over indicated time for each cell population measured by the MTT assay (bottom). (**B**-**D**) Immunofluorescence images (top) of subcellular localization of YAP (red) and TAZ (green) in cells expressing indicated shRNAs in U251 (**B**), GBM10 (**C**) and GBM14 (**D**) cells. Shown are maximum projection images. Scale bar, 15 μm. Staggered graphs (bottom) depict percentage of cells with YAP and TAZ in the cytosol (C), nucleus (N), or both (N/C). Number of cells analyzed per arm is shown above each bar. (**E**) U251 cells expressing indicated shRNA lentiviruses were transfected with either empty vector (Control) or FLAG-tagged YAP1-S127/S381A (YAP1-AA). Cell lysates were collected 2 days after transfection for Western blot analysis. Relative γH2AX levels are indicated. Same samples with equal loading amounts were run in parallel. (**F**) Graph shows relative luciferase activity (mean ± SEM, 3 biological replicates) of a YAP/TAZ responsive reporter (8xGTIIC) in LN229 cells expressing indicated shRNAs. Student's t-test, $^{**}P < 0.01$. (**G**) Immunoblot analysis of YAP1, TAZ and GAPDH in lysates from LN229 cells transduced with indicated shRNAs (top). Graph shown is representative (mean ± SD) of 3 biological repeats and presents cell viability over indicated time for each cell population measured by the MTT assay (bottom).

Supplemental Figure 6

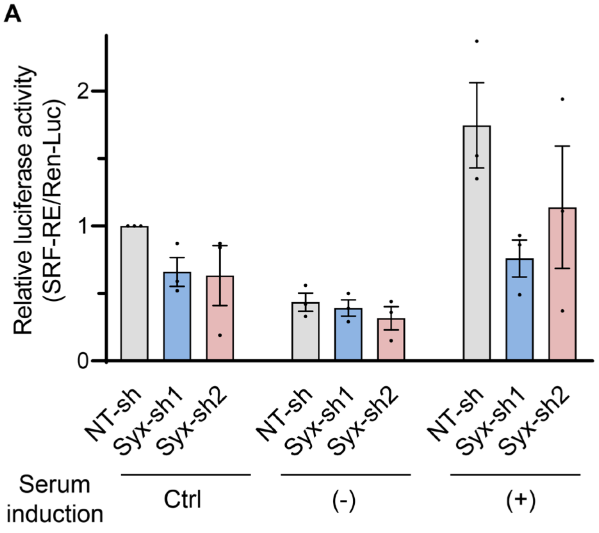

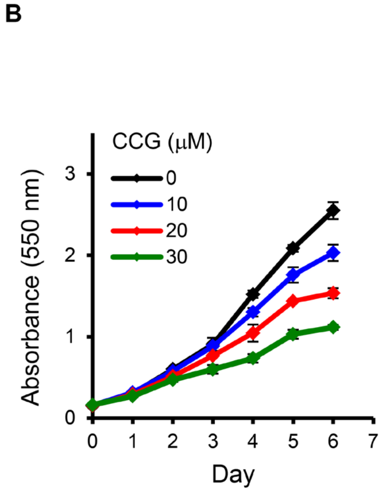

Supplemental Figure 6. Syx knockdown decreases SRF/MRTF signaling. (**A**) Graph shows relative luciferase activity of a SRF responsive promoter containing reporter (SRF-RE) normalized to Renilla luciferase (Ren-Luc) in U251 cells expressing indicated shRNAs. Bar graph depicts luciferase activity of cells grown in regular growth media (10% serum, Ctrl); under serum starvation for 18 h (-) and stimulated with 20% serum for 6 h after serum starvation (+). Bar graphs represent the mean ± SEM of 3 biological repeats with three technical replicates. (**B**) Representative (mean ± SD) graph of 2 biological repeats performed in triplicate, depicts U251 cell viability following treatment with different concentrations of the MRTF/SRF inhibitor CCG-203971 (CCG) for indicated times.

Supplemental Figure 7

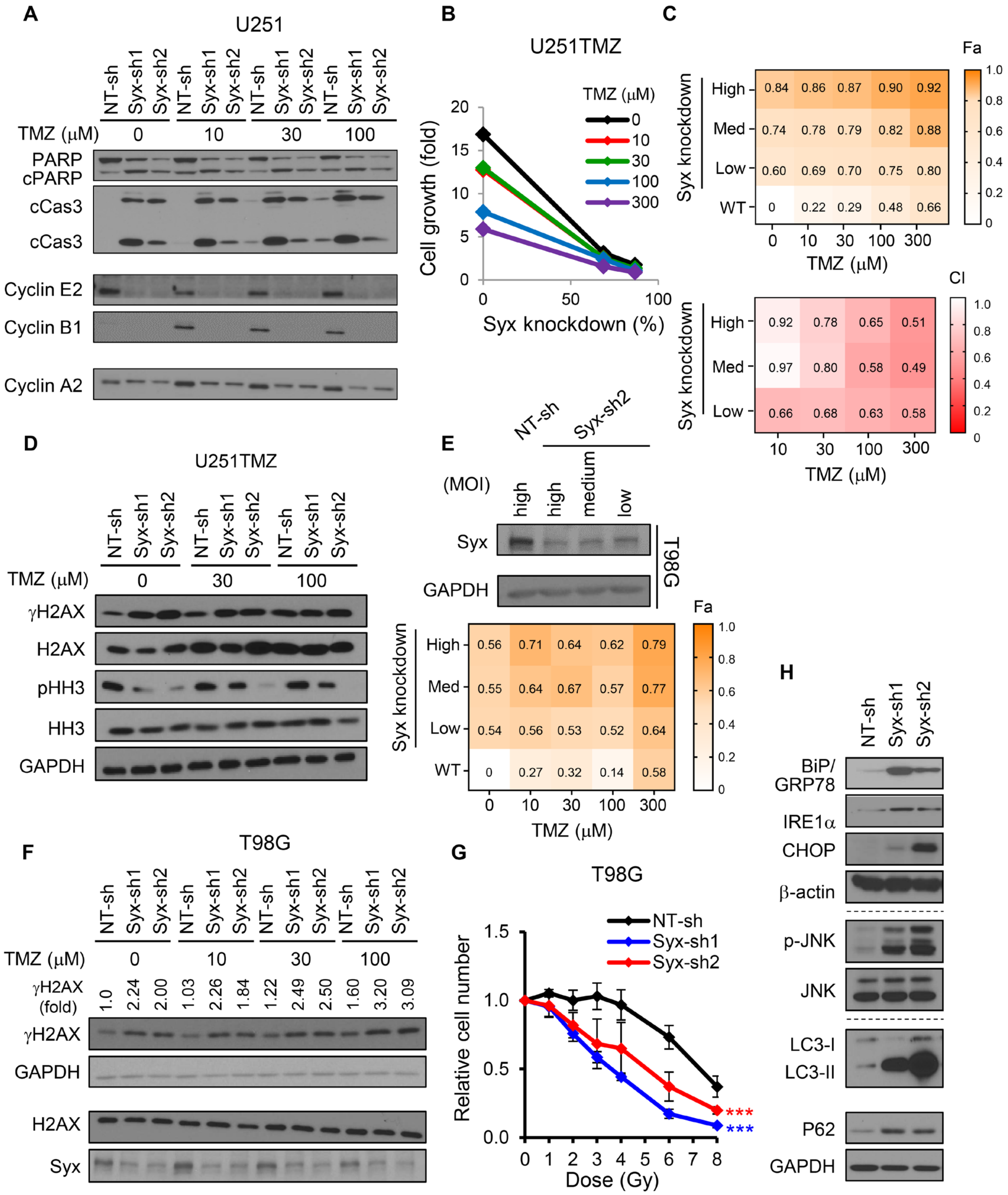

Supplemental Figure 7. Effects of Syx knockdown on TMZ resistance, ER stress and autophagy. (**A**) Immunoblot analysis of apoptosis effectors (cPARP, cCas3) and cyclins from U251 cells expressing indicated shRNAs treated with TMZ at different concentrations for 4 days. (**B**) Representative graph of 3 biological repeats depicts the growth of U251 TMZ resistant (U251TMZ) cells exhibiting different degrees (%) of Syx knockdown and treated with or without TMZ [0 μM (black), 10 μM (red), 30 μM (green), 100 μM (blue), 300 μM (purple)] for 5 days. (**C**) Heatmaps present average relative growth inhibition (top) and synergistic interaction (bottom) between Syx targeting and TMZ from 2-3 biological repeats. Different degrees of Syx knockdown were achieved using different amount of Syx-sh2 expressing lentiviruses (Low, 1% virus; Med, 2% virus; High, 4% virus). Fa, affected fraction. CI, combination index. Yellow to white scale (top) indicates high to low affected fraction, which corresponds to high to low growth inhibition (top). Red to white scale (bottom) indicates low to high CI, which corresponds to high to low synergy (CI < 1, synergy). (**D**) Immunoblot analysis of γH2AX, total H2AX, pHH3, total HH3 and GAPDH in lysates from U251 cells expressing indicated shRNAs treated with TMZ at different concentrations for 4 days. (**E**) Immunoblot analysis (top) from T98G cells expressing different amount of Syx-sh2 lentivirus. Heatmap depicts representative relative growth inhibition between Syx targeting and TMZ (3 biological repeats, bottom). (**F**) Immunoblot analysis from T98G cells expressing indicated shRNAs with 4-day TMZ. (**G**) Graph (mean ± SEM, 3 biological repeats) shows relative viability of T98G expressing indicated shRNAs following 8-day radiation treatment. Two-way ANOVA with Dunnett's multiple comparisons test, $***P < 0.001$ (**H**) Immunoblot analysis of ER stress markers (left) and autophagy markers (right) in lysates from U251 cells transduced with indicated shRNAs. For western blots (**A**, **D**, **E**, **F**, and **H**), different biological samples are separated by dashed lines. Same biological samples run at different times are indicated by larger white space. All other blots were run in parallel.
